# In tendons, differing physiological requirements lead to distinct patterns of MMP-1 degradation

**DOI:** 10.1038/s41598-025-32374-3

**Published:** 2025-12-23

**Authors:** Kelsey Y. Gsell, Laurent Kreplak, Samuel P. Veres

**Affiliations:** 1 School of Biomedical Engineering, Dalhousie University, Halifax, NS Canada; 2 Physics and Atmospheric Science, Dalhousie University, Halifax, NS Canada; 3 Division of Engineering, Saint Mary’s University, Halifax, NS Canada

**Keywords:** Collagen fibril, Enzymatic degradation, Collagenase, SEM, Tendon, Modeling, Biological techniques, Materials science

## Abstract

Collagen fibrils from high-stress, energy-storing tendons critical to locomotion are smaller in diameter with increased intermolecular crosslinking compared to fibrils from low-stress, positional tendons. This results in distinct loading mechanics thought to limit fatigue damage in energy-storing tendons. However, there appears to be a functional trade-off with energy-storing tendons also having reduced remodeling ability. Energy-storing tendons have lower collagen turnover and increased injury rates compared to positional tendons. In a recent study, a causative factor for this lower collagen turnover was suggested: resistance to degradation by MMP-1. To validate the prior study’s results obtained from single fibrils, the current study undertook population level assessment of fibril degradation by MMP-1. Predictive degradation models were created to assess fibril diameter distribution changes. Positional and energy-storing tendon sections were incubated for 24 h with buffer or MMP-1, imaged with scanning electron microscopy, and analysed with a custom pipeline for piece-wise fibril measurement. Enzyme treated sections showed evidence of degradation with reduced fibril diameter, decreased alignment, increased curvature, and decreased D-band length. Energy-storing tendon fibrils were more resistant to enzymolysis, with only the large diameter fibril subpopulation affected by MMP-1 (15% diameter reduction compared to control), while the entire population of positional tendon fibrils decreased in diameter (41%). Comparison to model predictions confirmed a linear relationship of degradation with fibril size. Larger fibrils experienced greater diameter decreases combined with increased longitudinal diameter variation and D-band decreases. Crosslinking is thought to be responsible for both fibril type and size findings, the latter suggesting higher density crosslinking in the fibril core.

**Supplementary Information:**

The online version contains supplementary material available at 10.1038/s41598-025-32374-3.

## Introduction

Collagen is the main structural protein in the body and is organized primarily into nanoscale fibrils. Collagen fibrils are long, rope-like structures that provide strength to connective tissues by resisting tensile forces and maintaining structural integrity under load. Fibril structure varies depending on the tissue type^1–3^, subtype^4,5^, and region^6^, with differences in protein composition, fibril size, molecular packing, and crosslinking. Even the characteristic D-band repeat—formed by the quarter-stagger packing arrangement of collagen molecules in the fibril—can vary (usually between tissue), but in tendon is typically 67 nm^2,7^.

Variations in collagen fibril structure strongly affect fibril mechanics. Molecular modeling and atomic force microscopy (AFM) studies have revealed that intermolecular crosslinks limit the sliding of adjacent collagen molecules within the fibril during tension-induced fibril elongation, affecting extension mechanisms and fibril plasticity^8–10^. Meanwhile, studies using scanning electron microscopy (SEM) have shown that intermolecular crosslinking affects susceptibility of collagen fibrils to structural damage during tensile overload^5,11,12^ and cyclic loading^5^.

While the dependence of mechanical functionality on collagen fibril structure is well established, the effect of variations in collagen fibril structure on biological processes critical to tissue homeostasis, remodeling, and healing are not well understood. In particular, whether collagen fibril structure affects enzymatic removal of undamaged collagen has yet to be thoroughly investigated. It is known that fibrils are protected against degradation by applied strain^13–16^, but the exact mechanism, i.e. the structural changes through which this occurs is unknown, as is the effect of differences in the structure alone. While cells are responsible for producing and releasing collagen molecules for assembly and enzymes for degradation, collagen itself is a mechanochemically sensitive structure that is able to influence assembly, growth, and degradation on its own^17^. Therefore, studying the fibril as a substrate, in addition to cellular mechanisms, is required to fully understand tissue homeostasis and repair.

Most studies that have attempted to directly assess the susceptibility of undamaged collagen fibrils to enzymatic degradation have used either non-physiologic enzymes^14,15,18^, non-native collagen fibrils^14,16^, or native fibrils from rodents^19,20^. Recently, we made direct assessment of the ability of the physiological collagenase (referring to the 3 mammalian MMPs capable of cleaving intact collagen fibrils) MMP-1, to remove collagen from native fibrils taken from both high and low-stress tendons in a large animal model^21^. The collagen fibril types used—bovine digital extensor tendon fibrils and superficial digital flexor tendon fibrils—have well documented differences in fibril size^5,22^, molecular packing^5^, collagen crosslinking^4,5,23,24^, and mechanical response^5,9^. Using AFM to image the same locations along individual fibrils both before and after enzyme exposure, we showed that both fibril size and tissue of origin affected fibril susceptibility to MMP-1, with fibrils from the high-stress, energy-storing flexor tendon showing significant resistance to degradation^21^. This finding supports in vivo measurements of lower collagen turnover in energy-storing compared to positional tendons^4,24^, despite them having greater cellularity^4,25,26^.

While providing the first indication that differences in collagen fibril structure between tissues may give rise to differential susceptibilities to MMP-1, the use of AFM to undertake very precise measurements of matching locations on fibrils before and after enzyme exposure made assessing a large number of fibrils unattainable. Therefore, these previous results do not adequately reflect the structural diversity among fibrils within each tendon type. In order to overcome this limitation and determine whether the prior results hold true at the fibril population level within tendon, the aim of the current study was to use SEM coupled with a custom, semi-automated image analysis pipeline to measure diameters of large numbers of collagen fibrils in tendon sections incubated with and without MMP-1. We hypothesized that, consistent with the results of our previous study^21^, both tissue of origin and fibril diameter would affect fibril susceptibility to MMP-1, with fibrils from the high-stress, energy-storing flexor tendon showing increased resistance to degradation and smaller diameter fibrils also showing increased resistance to degradation. To obtain additional insights into fibril size dependency of MMP-1 activity, mechanistic predictive modeling simulating fibril diameter distributions of whole tendon following different degradation scenarios was conducted. The modeling and experimental results confirmed that low-stress extensor tendons are composed of fibrils with significantly greater susceptibility to MMP-1 than those that make up high-stress flexor tendons, and that regardless of tendon type, smaller fibrils are more resistant to MMP-1 degradation than larger fibrils.

## Results

### Collagen fibrils from low stress tendons are more susceptible to degradation by MMP-1

**Fig. 1:**
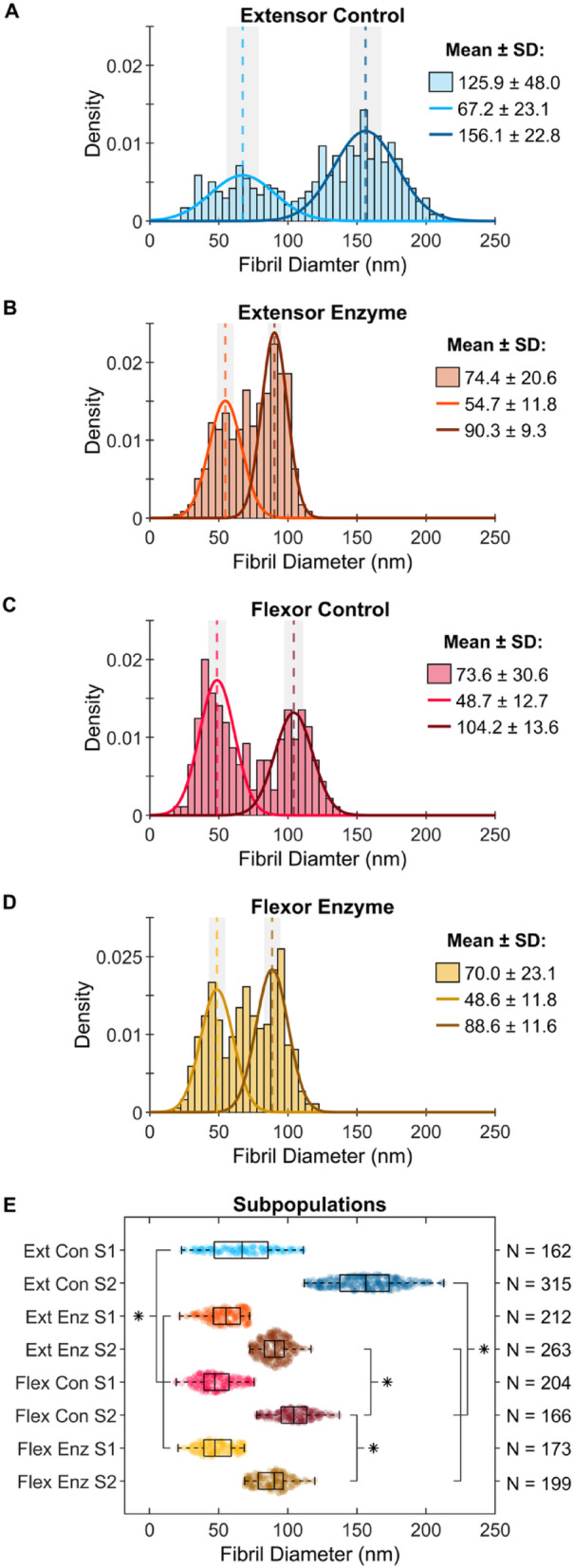
Average piecewise fibril diameter measured for bovine tendon sections incubated for 24 h in buffer alone (control) or buffer containing MMP-1 (enzyme). (**A**) The native bimodal distribution of control digital extensor tendon fibrils was drastically left-shifted in the (**B**) enzyme condition resulting in significantly decreased fibril diameters. (**C**) Control fibrils from digital flexor tendons were also bimodal with a more subtle, and non-significant left-shift in the (**D**) enzyme treated sample. (**E**) Following enzyme incubation, fibril subpopulations were significantly smaller in both extensor tendon subpopulations, while only the large diameter flexor fibrils significantly decreased compared to control (S1 is smaller subpopulation, S2 larger subpopulation). While for control samples the fibril subpopulations between tendons were significantly different, they converged to statistically similar diameters following degradation.

Tendon type influenced the ability of MMP-1 to remove material from collagen fibrils. Average fibril diameter was significantly smaller in bovine extensor tendon sections incubated with MMP-1, compared to the control condition of incubation in buffer alone (Fig. 1B vs. Fig. 1A; *p* < 0.0001). No difference between conditions was detected for bovine superficial flexor tendon sections when comparing the full diameter distribution (Fig. 1C, D; *p* = 0.1362). There was evidence of flexor fibril degradation, however: by examining the flexor tendon fibril subpopulations (Fig. 1E), the large diameter subpopulation had significantly smaller fibrils in the enzyme treated group compared to control (*p* = 0.0002), while no difference was detected between the small diameter sub-populations (*p* = 1.000). Both subpopulations in the extensor tendon showed significantly smaller diameters in the enzyme sample compared to control (*p* = 0.0013 and *p* < 0.0001 for the small and large subpopulations, respectively).

Both flexor and extensor tendons exhibited a native bimodal distribution of fibril diameters (Fig. 1A, C), as previously reported by Herod et al.^5^. Small and large fibril diameter subpopulations for both extensor and flexor tendons were statistically similar between Herod et al.^5^ and the control data from the current study (*p* = 1.000 for all four groups). While the subpopulations were statistically different between extensor and flexor tendons (*p* < 0.0001 for both), both bimodal distributions shifted towards lower diameters (shift to left) with MMP-1 incubation. The extensor tendon exhibited a dramatic shift with the entire distribution moving to the left, completely displacing the large fibril sub-population peak, while the flexor shift was more subtle yet quantifiable. Interestingly, the distributions for the sections that were incubated with MMP-1 appeared to be similar between the two tendons, and indeed fibril subpopulations in extensor and flexor samples incubated with MMP-1 were statistically undistinguishable from each other (*p* = 1.000 for both).

### Smaller fibrils are more resistant to degradation by MMP-1

**Fig. 2:**
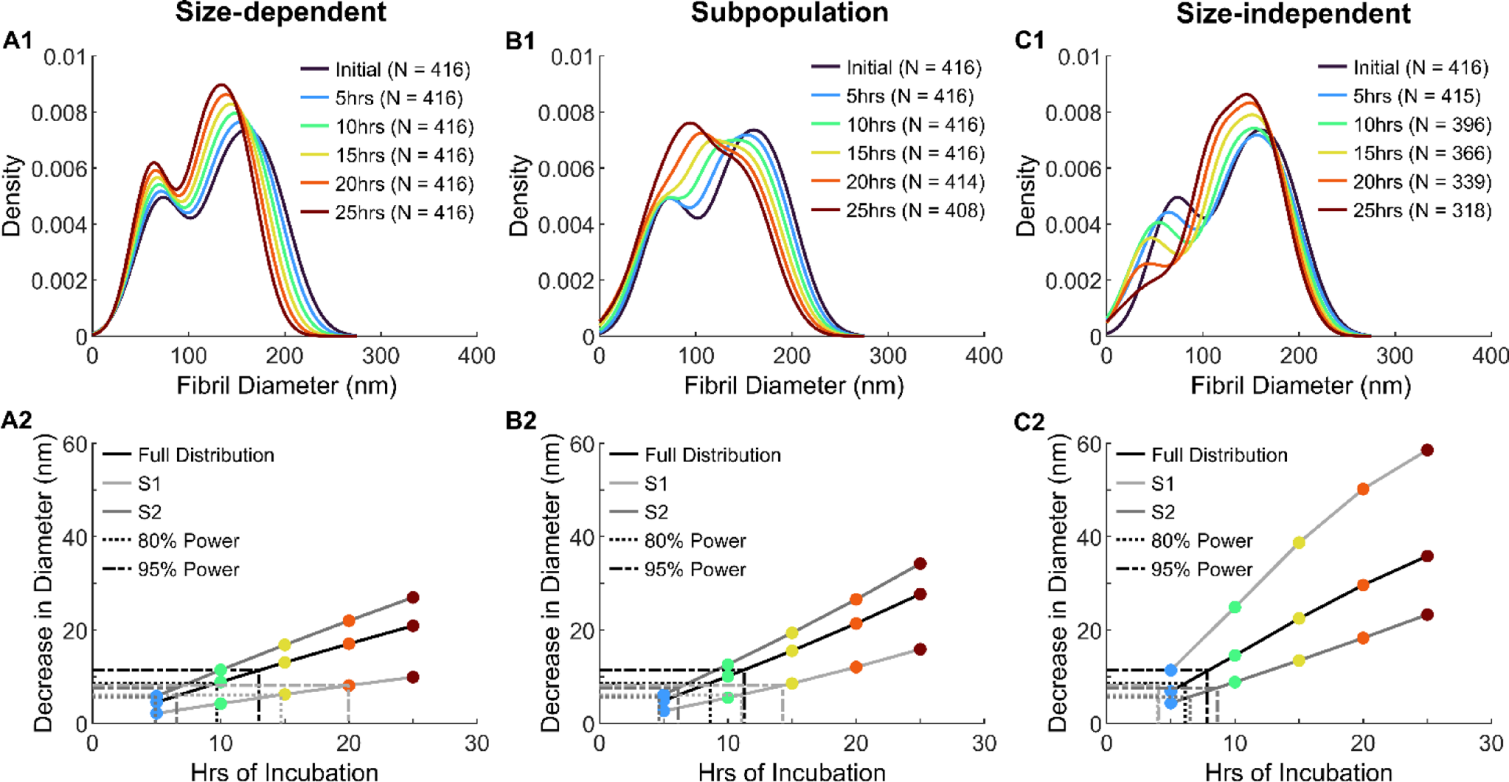
Modeled change in collagen fibril diameter with MMP-1 incubation between 5 and 25 h. Initial population of fibril diameters taken from^5^ with change in diameter due to degradation based on data from^21^. Three unique models of degradation are shown. (**A**) Size-dependent degradation, (**B**) subpopulation degradation, and **C**) size-independent degradation. 1) Kernel fit of density histograms with 2) corresponding cumulative decrease in fibril diameter are shown, with the latter visualizing the full distribution of fibril diameters, small diameter fibril subpopulation (S1), and large diameter fibril population (S2). Dotted lines indicate diameter change able to be detected with (··) 80% and (- ·) 95% power.

**Fig. 3:**
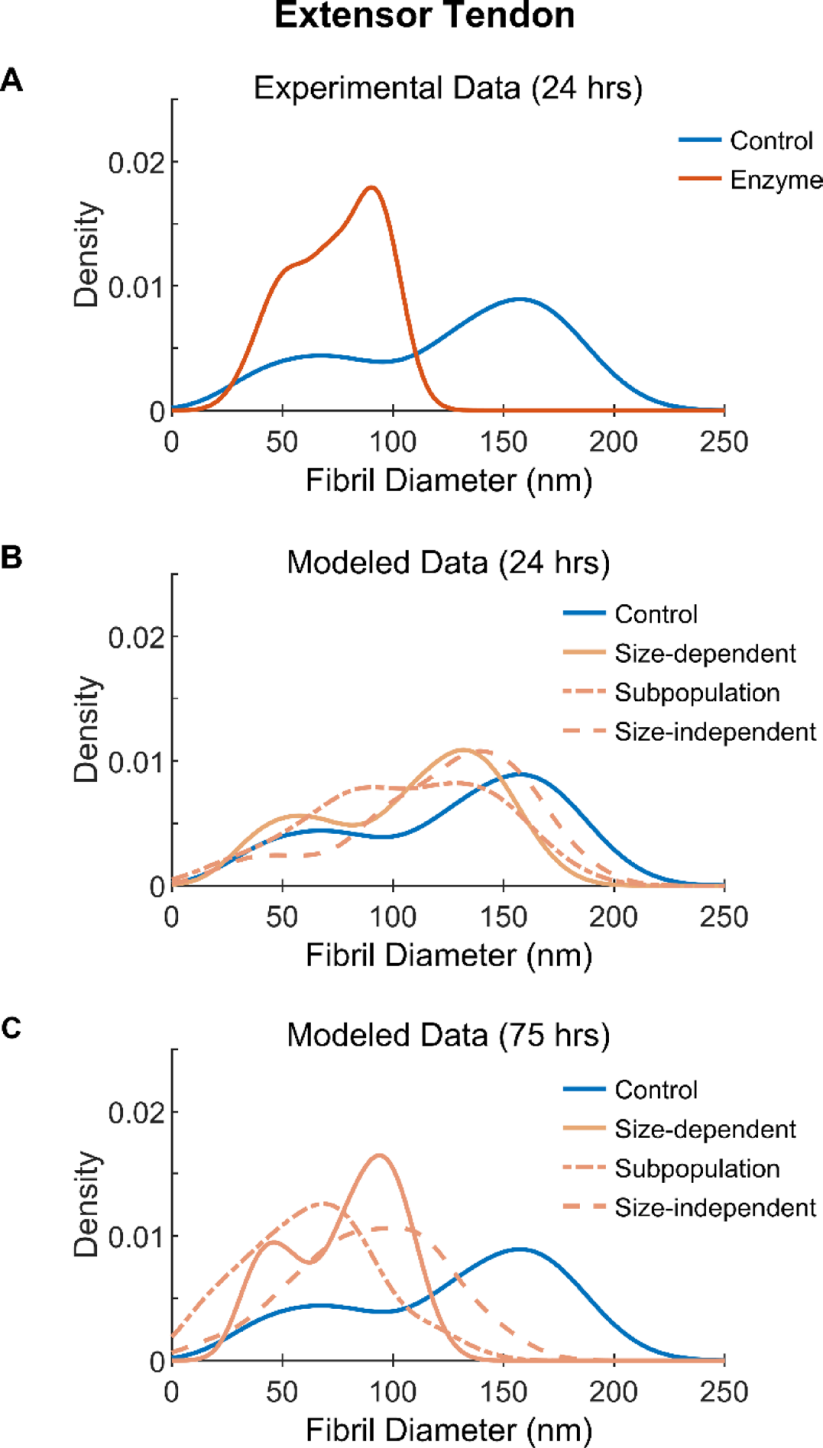
Collagen fibril diameter distributions with experimental and modeled MMP-1 degradation of bovine digital extensor tendons. (**A**) Experimental data from control and enzyme conditions after 24 h of incubation in buffer alone or buffer containing MMP-1, respectively. (**B**) Degradation modeled in three ways using experimental control data and equations derived from previous work on single fibrils^21^ with 24 h and (**C**) 75 h of incubation.

Collagen fibril diameter was shown to affect the ability of MMP-1 to remove material from extensor fibrils. Using the magnitude of degradation observed in our previous study^21^, our mechanistic predictive models of extensor tendon degradation revealed distinct fibril diameter distribution shapes when fibril size was accounted for (Fig. 2). The size-dependent model of degradation (Fig. 2A1), which implemented change in cross-sectional area (CSA) for each fibril individually, preserved the native bimodal shape of the diameter distribution throughout the 25 h of modeled incubation. Both peaks shifted left with increases in peak amplitude, as removal of material was proportional to fibril size (Fig. 2A2). This was similar to what was measured in the experimental degradation data from the present study (Figs. 1A,B and 3A). However, the experimentally observed left shift of the distribution far surpassed that of the modeled size-dependent degradation shown in Fig. 3B. When extending the modeled incubation time to 75 h (Fig. 3C), the location and magnitude of the subpopulation peaks with the size-dependent model better approximated the experimentally observed outcome after 24 h of incubation. The other two degradation models were poor predictors of the experimentally observed degradation results as the two subpopulation peaks converged over time with the subpopulation model of degradation (Fig. 2B1) losing the large diameter subpopulation peak and the size-independent model (Fig. 2C1) losing the small diameter subpopulation peak. These results suggest that MMP-1 degradation of extensor collagen fibrils is size-dependent, with larger fibrils being more susceptible to degradation than smaller fibrils.

**Fig. 4:**
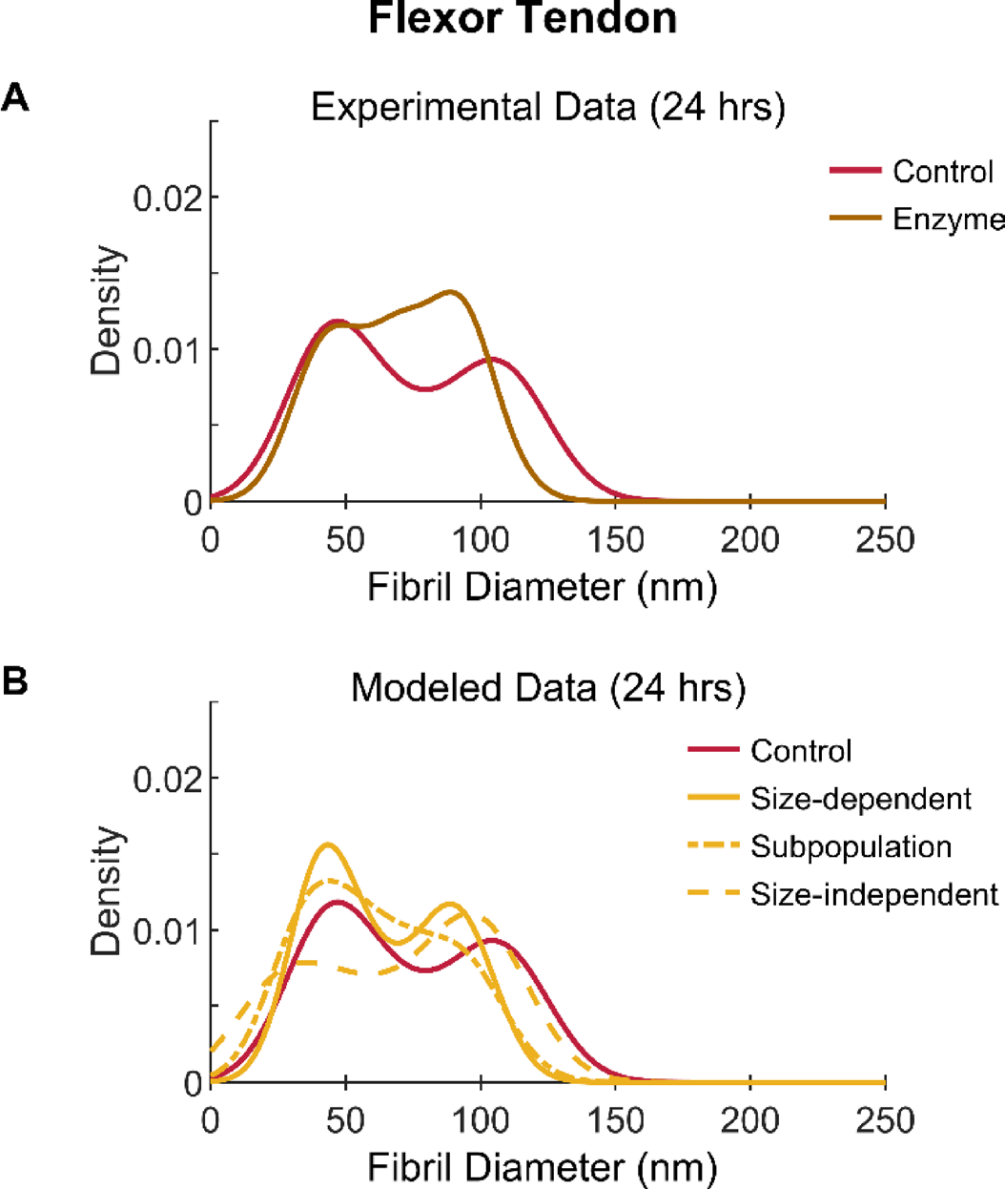
Collagen fibril diameter distributions with experimental and modeled MMP-1 degradation of bovine digital flexor tendons. (**A**) Experimental data from control and enzyme conditions after 24 h of incubation in buffer alone or buffer containing MMP-1, respectively. (**B**) 24 h degradation modeled in three ways using experimental control data and equations derived from previous work on single extensor fibrils^21^.

To attempt to uncover the mechanism of flexor fibril degradation, the fibril diameters from the flexor control sample were also entered into the extensor-based degradation models (Fig. 4). Unlike the extensor tendon, the amplitude of the experimentally observed flexor degradation was similar to that of the modeled degradation after 24 h. However, the shape/peak location of the modeled distributions did not fit the experimental results as clearly as for the extensor case suggesting a more complex relationship.

### Fibril size influences diameter variation along the fibril and D-band length after MMP-1 degradation, even in the high-stress flexor tendon.

**Fig. 5:**
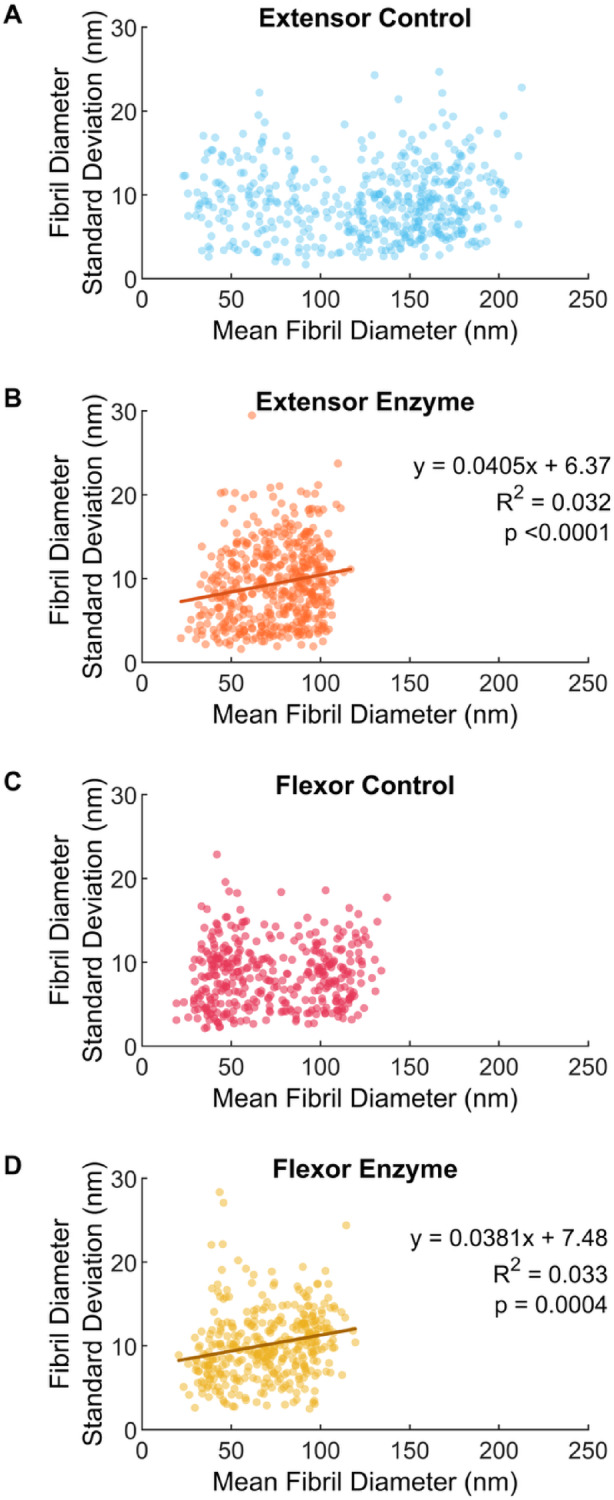
Variation of piecewise fibril diameter along the fibril length (measured as standard deviation) increased with average piecewise fibril diameter after degradation by MMP-1. (**A**) Bovine extensor tendon section incubated in buffer alone and (**B**) buffer containing MMP-1. (**C**) Bovine flexor tendon section incubated in buffer alone and (**D**) buffer containing MMP-1.

Examination of other outcome measures suggests size-dependence with degradation indeed exists for both tendons. The variation in diameter along the fibrils, quantified using the standard deviation in piecewise fibril diameter was size dependent in the enzyme condition (Fig. 5B, D). Fibrils that were incubated in MMP-1 displayed a significant positive linear relationship for both the extensor (*p* < 0.0001) and flexor tendon (*p* = 0.0004) between longitudinal variation in diameter and average diameter. The control conditions were not influenced by diameter (Fig. 5A,C; *p* = 0.4159 and 0.2591, respectively). Therefore, larger fibrils after degradation by MMP-1 had more variation in diameter along their length. This relationship was similar between tendons (*p* = 0.9921). While there was no difference in mean diameter variability between control and enzyme samples in extensor tendons (*p* = 0.9065), there was in the flexor tendon. Flexor fibrils degraded by MMP-1 had greater diameter variability compared to flexor control fibrils (*p* < 0.0001), but also compared to extensor fibrils incubated with MMP-1 (*p* = 0.0336).

**Fig. 6:**
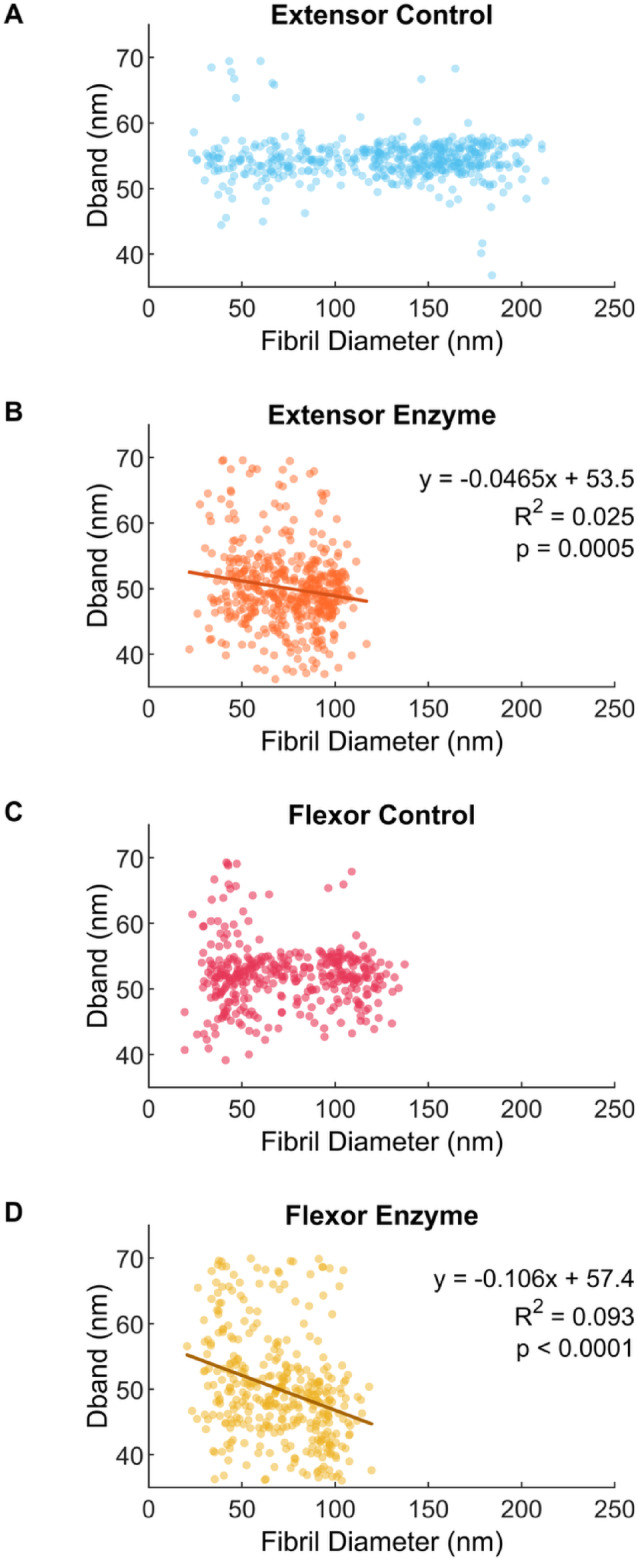
D-band length decreases with larger fibril diameter after degradation by MMP-1. (**A**) Bovine extensor tendon section incubated in buffer alone and (**B**) buffer containing MMP-1. (**C**) Bovine flexor tendon section incubated in buffer alone and (**D**) buffer containing MMP-1.

**Fig. 7:**
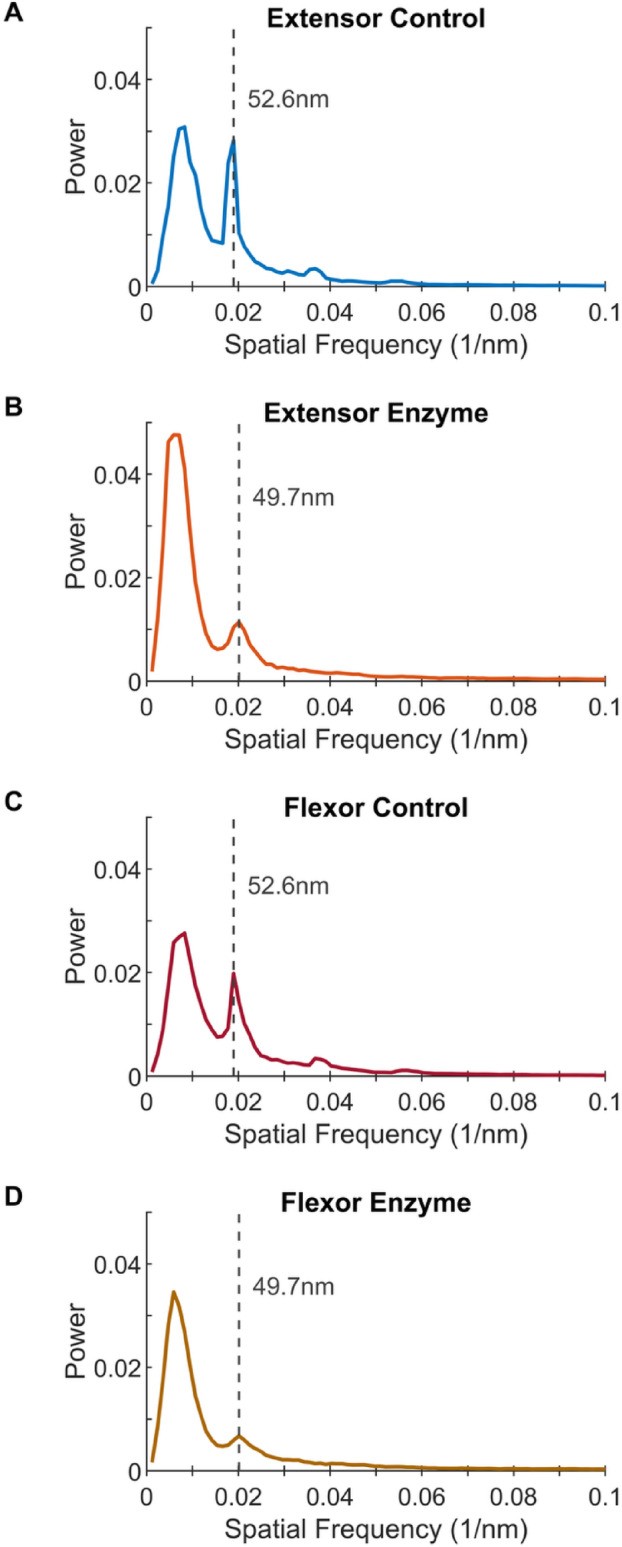
Averaged fibril spatial power spectra for each of 4 tendon section samples, reveals D-band length decreases with MMP-1 degradation. (**A**) Bovine extensor tendon section incubated for 24 h in buffer alone (control) and (**B**) buffer containing MMP-1 (enzyme). (**C**) Bovine flexor tendon control and (**D**) enzyme sections. Spatial frequency corresponding to D-band length is indicated with a gray dashed line.

Fibril size also influenced D-band length change with degradation (Fig. 6). For both tendons, fibril D-banding was significantly shorter after degradation by MMP-1 (*p* < 0.0001 for both), with increased shortening in larger fibrils. Fibrils that were incubated in MMP-1 displayed a significant negative linear relationship for both the extensor (*p* = 0.0005) and flexor (*p* < 0.0001) tendon while control fibril D-band lengths were not influenced by diameter (*p* = 0.3373 and 0.4068, respectively). The relationship between diameter and D-band was different between tendons (*p* = 0.0054), with a greater negative relationship in the flexor fibrils. To better account for the high variation in individual fibril D-bands, particularly in the enzyme treated fibrils which also produced lower spatial signal power, the power spectra of all fibrils within each sample were averaged, rather than comparing the average fibril D-bands. Figure 7 shows that the D-band peak power decreases with enzyme incubation with an average D-band length of 52.6 nm in flexor and extensor control samples, and smaller D-band length of 49.7 nm in the MMP-1 treated samples. This is an almost 6% reduction in D-band spacing due to enzyme incubation.

### Incubation with MMP-1 resulted in disorganization of collagen fibrils in both tendons

**Fig. 8:**
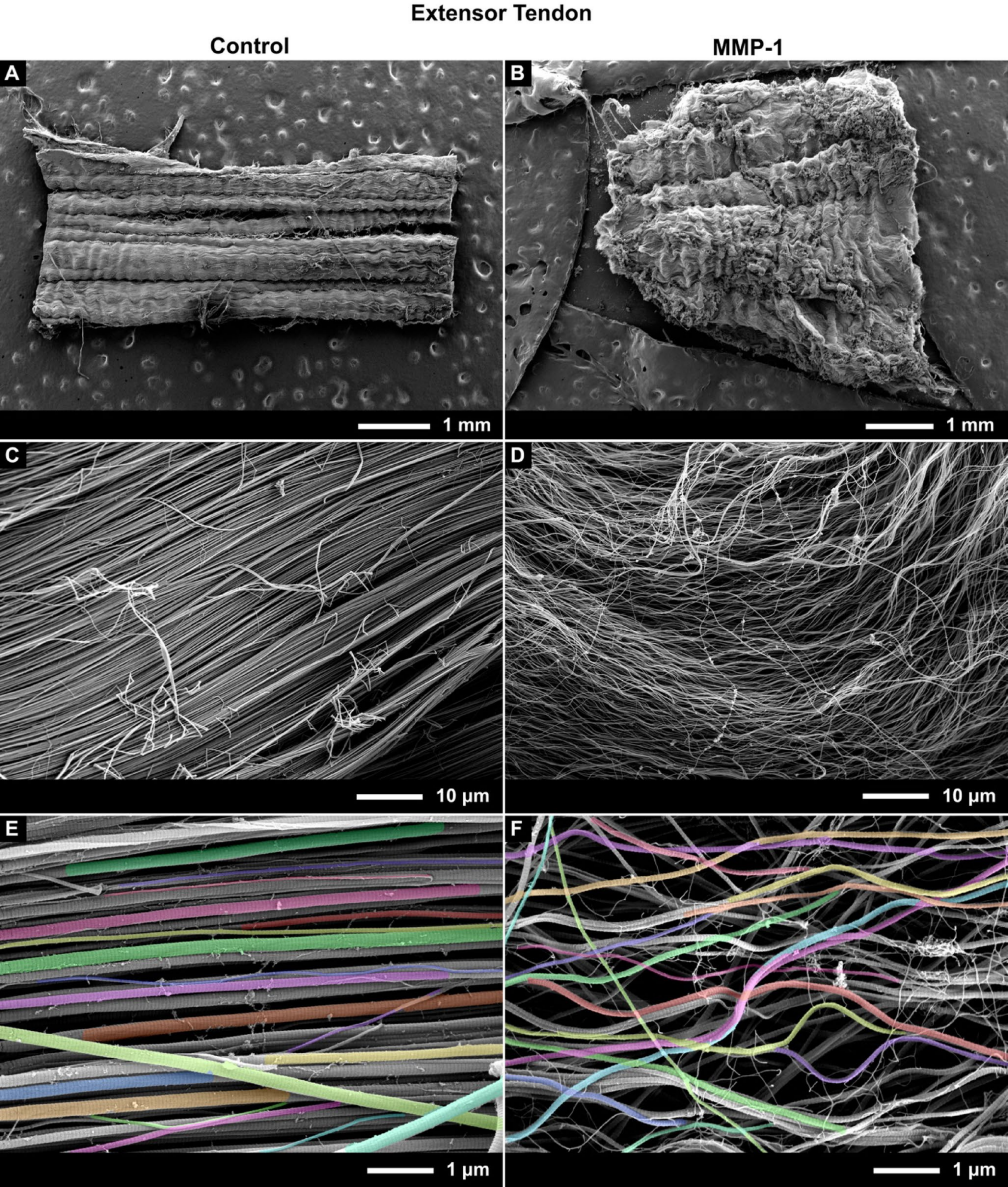
Representative SEM images of bovine extensor tendon sections incubated for 24 h with buffer only (Control) (Left: **A**, **C**, **E**) or with buffer containing MMP-1 (Right: **B**, **D**, **F**). From top to bottom, images are shown at ~ 50, 1.5 k, and 15kX magnification. Images at 15kX were used for image analysis, with colored fibrils indicating segmentation.

**Fig. 9:**
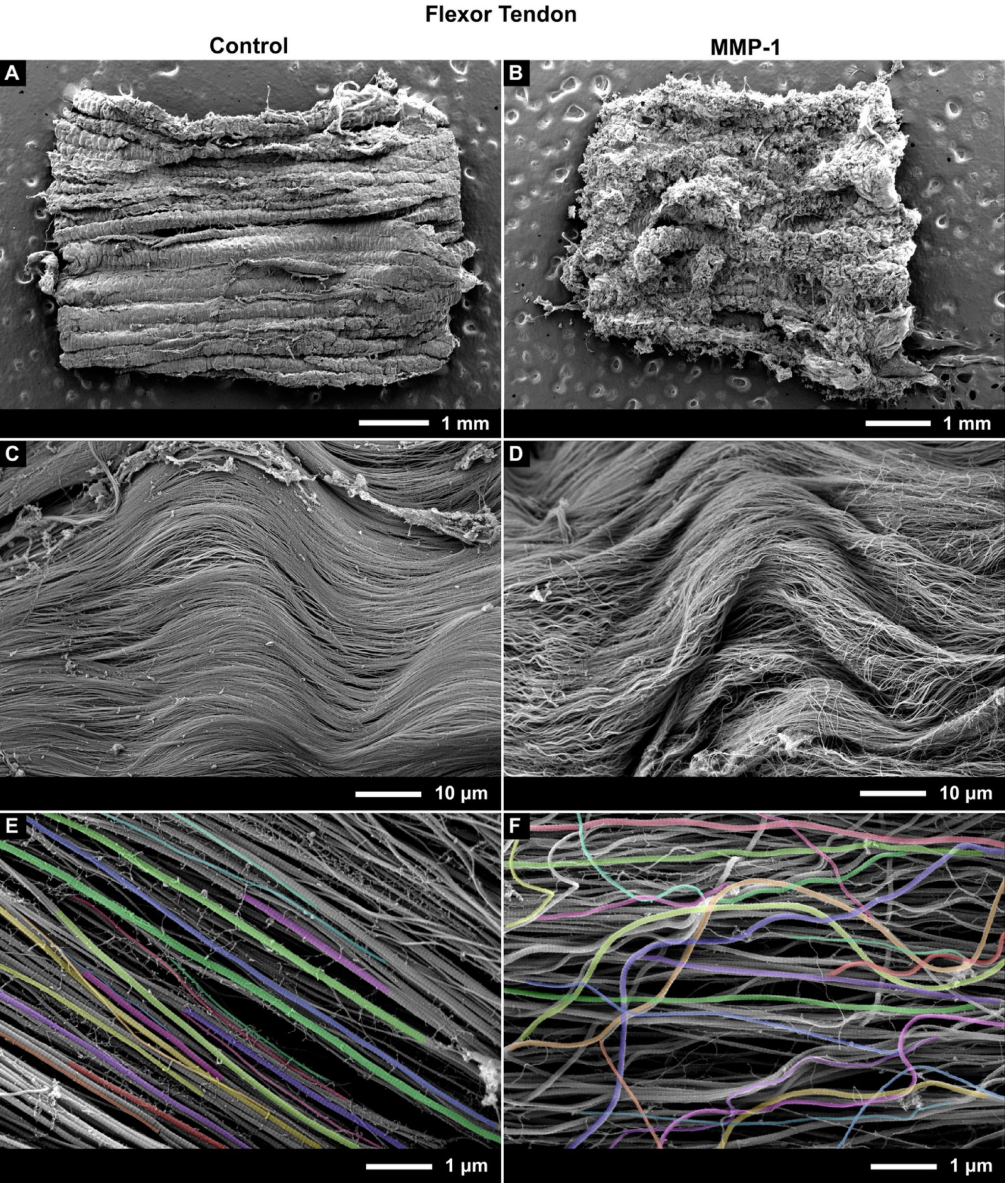
Representative SEM images of bovine flexor tendon sections incubated for 24 h with buffer only (Control) (Left: **A**, **C**, **E**) or with buffer containing MMP-1 (Right: **B**, **D**, **F**). From top to bottom, images are shown at ~ 50, 1.5 k, and 15 k X magnification. Images at 15kX were used for image analysis, with colored fibrils indicating segmentation.

Tendon sections from both extensor and flexor tendons showed visible structural differences after 24 h of degradation with MMP-1, becoming more disorganized, which was quantified by measuring fibril alignment and curvature. From a macroscopic perspective, the disorganisation of the tendons incubated with MMP-1 could be seen by eye compared to sections from the same tendon sample incubated with buffer alone (control) (Fig. 8B vs. Fig. 8A and Fig. 9B vs. Fig. 9A). Upon increasing magnification in MMP-1 incubated samples the fibrils were less tightly packed and had taken on a wavy morphology as opposed to being tightly aligned as in the control samples (Fig. 8D vs. Fig. 8C & Fig. 9D vs. Fig. 9C).

**Fig. 10:**
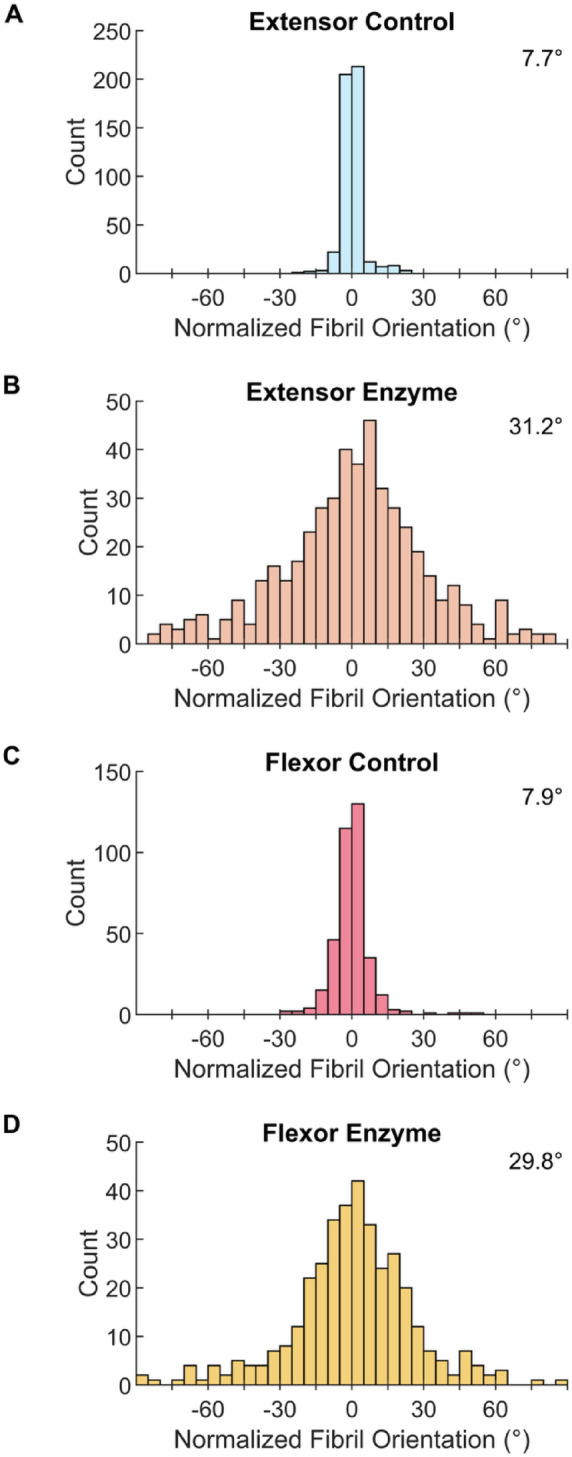
Collagen fibril alignment, measured by variation of fibril orientation angle, decreases with MMP-1 degradation. (**A**) Bovine extensor tendon section incubated for 24 h in buffer alone (control) and (**B**) buffer containing MMP-1 (enzyme). (**C**) Bovine flexor tendon control and (**D**) enzyme sections. SD is indicated on figure.

**Fig. 11:**
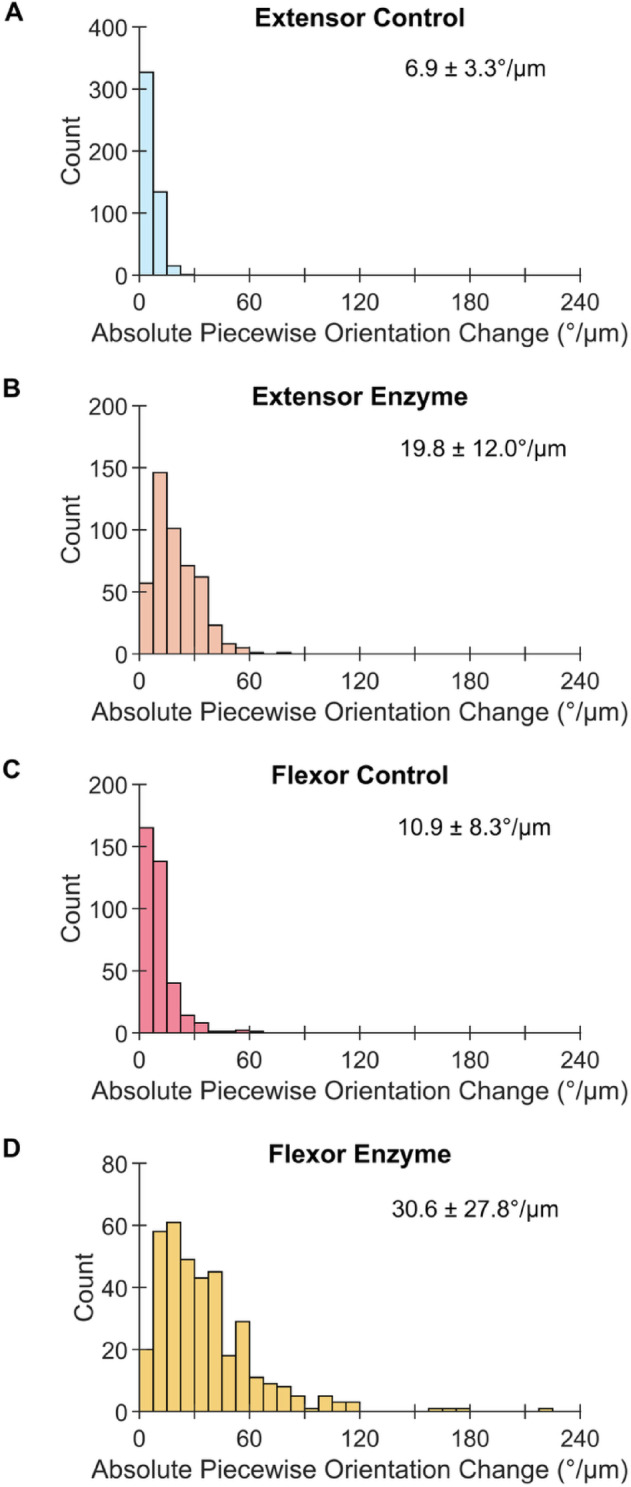
Collagen fibril curvature increases with MMP-1 degradation. (**A**) Bovine extensor tendon section incubated for 24 h in buffer alone (control) and (**B**) buffer containing MMP-1 (enzyme). (**C**) Bovine flexor tendon control and (**D**) enzyme sections. Mean ± SD are indicated on figure.

The increased waviness of fibrils following MMP-1 incubation was also apparent at the high-magnification (15kX) used for image analysis (Fig. 8E vs. Fig. 8F and Fig. 9E vs. Fig. 9F). Disorganization was quantified using measures of fibril alignment and curvature, both of which changed significantly with enzyme incubation in both tendons (Figs. 10, 11). The variance in fibril alignment, or normalized orientation (per image), was significantly greater in both tendons for sections incubated with MMP-1 compared to controls (*p* = 0.0004 for both). Fibril alignment was not different between tendons in either incubation condition (*p* = 0.4356 and 0.6556 for control and enzyme conditions respectively). Fibril curvature increased with enzyme incubation for both tendons (*p* < 0.0001 for both), but flexor fibrils had greater curvature than extensor fibrils in both conditions (*p* < 0.0001 for both).

## Discussion

The aim of the current study was to design and carry out an experiment with the statistical power and physiologic conditions required to validate that collagen fibrils from low-stress positional tendons are more susceptible to enzymatic degradation by MMP-1 than high-stress energy-storing tendons. This was confirmed through the direct measurement of structural changes: namely a decrease in size due to incubation with MMP-1. Additionally, the influence of fibril size on degradation was validated through the creation and comparison of mechanistic predictive models of degradation with experimental results. This work presents both novel findings and experimental/methodological procedures.

### High-stress energy-storing tendons are less susceptible to MMP-1 degradation than low-stress positional tendons

The well-established large animal forelimb model, consisting of the digital extensor and flexor tendons^4,5,9,11,23–26^, was used to examine the effects of different fibril structures on enzymatic degradation. Extensor tendons are considered low-stress positional tendons owing to their function of positioning the skeleton, while flexor tendons similar to the human Achilles, are classified as high-stress and energy-storing due to much higher in vivo maximum stress and their ability to store and return elastic strain energy which aids in movement^4,27^. These functional differences coincide with multiscale structural differences, including at the fibril level. Flexor tendons have smaller diameter fibrils^5,22^, and greater molecular packing and crosslinking^4,5,23,24^. While the greater crosslinking of the flexor fibrils is thought to limit fatigue damage during cyclic loading^5,9^, the current study provides evidence that crosslinking also appears to limit the ability for fibrils to be degraded and potentially remodeled. This points to substrate specific effects that support the lower collagen turnover seen in energy-storing tendons compared to positional tendons^4,24^ despite energy storing tendons possessing greater cellularity^4,25,26^.

Our previous study used AFM to measure the cross-sectional area (CSA) of isolated collagen fibrils before and after incubation with MMP-1 and found that the more crosslinked flexor fibrils showed no evidence of degradation by way of CSA decrease after 5 h while the less-crosslinked extensor fibrils did^21^. While that study was the first to directly quantify degradation using single fibrils and a physiological collagenase, it was limited to a small sample size (n≈15 for each group). Isolation of single fibrils and adherence to a substrate also resulted in shape changes with drying, inaccessibility of enzyme to the entire fibril surface, and a non-physiologic environment (i.e. fibrils in isolation rather than packed into fibers).

The present study overcame those limitations by using tendon sections. SEM allowed for rapid imaging of hundreds of fibrils, providing the statistical power to compare fibril diameters at the population level through the use of paired control and enzyme samples from the same location in each tendon. Additionally, a custom semi-automated image analysis pipeline was used to measure not only average fibril diameter, but the variation in diameter along fibrils, fibril alignment and curvature, and D-band length, all measures which may be affected by enzymatic degradation.

Collagen fibrils from the bovine extensor tendon—the low stress, positional tendon—were clearly degraded after 24 h incubation with MMP-1. These fibrils had noticeably smaller diameters than control fibrils, which was visible even by eye at high magnification (Fig. 8E vs. Fig. 8F). When quantified using the piecewise image analysis pipeline, the average fibril diameter for the extensor tendon decreased by ~ 41% compared to control, while the small and large diameter fibril subpopulations saw decreases of ~ 19 and 42%, respectively, all of which were statistically significant.

Collagen fibrils from bovine flexor tendons—the high-stress energy-storing tendon—were not as clearly degraded as the extensor fibrils. There was no significant change in the average fibril diameter (~ 5%) or the average of the small diameter fibril subpopulation (~ 0%), but the large diameter population saw a significant decrease of ~ 15%. It is also important to note that the “small” and “large” diameter clusters in the two different tendons are centered around different mean diameters. Extensors have populations at 67.2 ± 23.1 nm and 156.1 ± 22.8 nm, while the flexors have populations at 48.7 ± 12.7 nm and 104.2 ± 13.6 nm.

In addition to changes in fibril diameter, sections from both tendons showed further evidence of degradation. On the macroscale the sections appeared more wavy, likely due to the relaxation/elastic recoil as the interfascicular matrix components were degraded or disconnected from the fibrils. This also led to less defined fiber and fascicle structures as the fibrils were less constrained, evident by decreased packing. On the nanoscale this resulted in a measurable decrease in fibril alignment and increase in fibril curvature. Typical of tendon fibrils, control samples from both tendons were highly aligned with only ~ 8° of deviation in mean orientation angle. This increased to ~ 30° of deviation in MMP-1 treated samples. While alignment was similar between tendons in both conditions, fibril curvature was higher in flexor fibrils compared to extensors in both conditions. This makes it difficult to make conclusions about differences in curvature between tendons as a result of degradation. Because tendon differences existed in both control and enzyme conditions, this could in part be due to the fact that flexor fibrils are smaller than extensor fibrils, making them more flexible when tension is released (smaller bending rigidity) leading to increased curvature. Samples were unconstrained/not under tension during the incubation, with further relaxation occurring after degradation. Additionally, energy-storing flexor tendons have tighter crimp and higher elastin content^5,28–30^, resulting in greater relaxation than in extensor due to larger elastic recoil; the crimp being released could have resulted in more wavy fibrils.

Fibril D-band length also decreased by ~ 6% with enzymatic degradation based on the peak locations from the averaged power spectra of each condition. Decreasing D-band length has been reported with degradation of an un-fixed, wetted section of cornea by bacterial collagenase (BC)^18^, however with ~ 25% decrease in D-band length. We propose that enzymatic cleavage releases fibril pre-strain: the internal mechanical strain generated by the axial twist of molecules within the fibril^19,31^. BC cleaves at multiple locations along the collagen molecule which should result in more release of pre-strain, and a greater reduction in D-band (with a corresponding decrease in diameter of ~ 30%). In contrast, MMP-1 cleaves only at the canonical MMP cleavage site but was still able to yield a larger relative reduction in diameter (~ 40%).

Also of note are the average D-band periods reported in the present study. D-band periods for control samples (~ 53 nm) were noticeably smaller than the often-referenced value of 67 nm for tendon. While initially surprising, it was quickly evident that this is because the majority of SEM studies do not quantify D-band period. However, using x-ray diffraction measurements Fullwood and Meek reported that the preparation of samples for electron microscopy results in changes to collagen spacing (intermolecular, interfibrillar, and D-period)^32^. In their study, glutaraldehyde-fixed bovine cornea fibrils (37.4 ± 1.4 nm in diameter) were found to undergo a large decrease in D-period from ~ 65 nm in fresh cornea to between 52 and 57 nm after critical point drying. This would suggest the values measured in the present study are realistic and should serve as a strong reminder that for samples imaged with SEM, any investigation of fibril D-band should always be compared to a control sample when prepared with critical point drying.

### Smaller diameter collagen fibrils are more resistant to degradation by MMP-1

Our previous study also discovered a size effect to degradation, where larger extensor fibrils were degraded more than smaller ones^21^. We set out to model what degradation with this size effect would look like at the population level, termed the size-dependent model, and included models where the size-dependence was a result of two different subpopulation degradation rates and where degradation was completely size-independent (one rate). By applying these predictive models first to a reference dataset of fibril diameters from bovine extensor tendons^5^ (Fig. 2), then to the extensor control data from the current study (Fig. 3), it was clear that degradation was indeed size-dependent.

The change in extensor fibril diameter distribution over time with each of the three degradation models yielded dramatically different results. The size-dependent model was the only model that retained the native bimodal distribution shape, which was clear in both the control and enzyme samples. However, the magnitude of degradation in the experimental incubation with MMP-1 was much greater than that which was modeled. By increasing the modeled incubation time to 75 h the magnitude of degradation more accurately reflected that which was observed experimentally.

The degradation models were based on data from the previous AFM study^21^. At the full population level, the average degradation after 5 h of incubation for the extensor tendon was modeled to be 4.8 nm (all three models; Fig. 2A2, B2, C2). This would equate to roughly 1 layer of molecules removed from the surface of the fibrils. If this degradation rate had been maintained over the 24 h, an average decrease of ~ 20 nm would have been expected, but experimentally a decrease of 52.3 nm was observed. This suggests a ~ 2.6 times increase in the expected degradation of the extensor fibrils.

Larger fibrils being degraded more than smaller fibrils was not simply due to the greater substrate availability of the enzyme by way of increased surface area of larger fibrils. If this relationship was due to the difference in surface area alone, all fibrils, regardless of size would experience a similar decrease in absolute diameter. This is because larger fibrils require more collagen molecules to be removed to strip a single layer. However, larger fibrils would have a smaller relative decrease in diameter compared to their initial diameter. In our data, neither is true; both the absolute and relative decrease in diameter is greater for larger fibrils.

An example of this can be seen by looking at the location of the subpopulation peaks in the extensor data (Fig. 1A, B). The small diameter peak (67.2 nm) decreased by 12.5 nm, or ~ 19%. If degradation was due only to differences in surface area, the large diameter peak would also have decreased 12.5 nm, or 8%. Instead, the large diameter peak decreased by 65.8 nm or 42% of the initial diameter. This is much greater than surface-peeling can explain.

Flexor fibril degradation also appears to be influenced by fibril size. Our previous AFM study did not detect degradation of the flexor fibrils after 5 hrs^21^. By increasing the incubation to 24 h in the current study, degradation was seen, but for the large diameter fibril subpopulation only. This suggests some size dependence. Indeed, when examining other structural features with average diameter as a covariate, there were significant linear relationships with fibril size. Both extensor and flexor fibrils that were degraded with MMP-1 had greater variation in diameter (Fig. 5) and greater decrease in D-band length (Fig. 6) for larger diameter fibrils, likely indicative of increased degradation.

### *Greater intrafibrillar crosslinking inhibits enzymatic degradation *via* MMP-1*

While differences in crosslinking are thought to be the reason for the different tendon-dependent degradation rates observed in the current study, we suggest that crosslinking is also the cause of the size-dependent degradation finding in both tendons. Crosslinking is thought to inhibit the accessibility of MMP-1 to its binding and cleavage site^33^. Flexor fibrils have greater density of crosslinking and increased mature trivalent crosslinks compared to extensor tendons^4,5,23,24^, with this divergence forming prenatally^34^. The latter discovery was made in a recent thesis from our research group, where similar measures of crosslink density, represented by the time of load decay during thermal relaxation under load, were found in the third-trimester fetal bovine extensor tendons^34^ as those reported for the same tendon in adulthood^5^. This implies that while positional tendon fibril diameters increase from birth to skeletal maturity^22,34,35^, the density of crosslinks do not change. Other work looking at crosslinking changes over the adult lifetime in mouse tail tendon, a positional tendon, has found that while immature crosslinks relative to amount of collagen decreases with age, they are not all replaced by mature crosslinks^36^. This suggests that collagen fibrils from the adult positional tendon may have a more crosslinked core compared to the periphery. A more crosslinked fibril core would explain why MMP-1 degradation was found to be size-dependent in both our previous study using single collagen fibrils^21^ and in the current investigation.

**Fig. 12:**
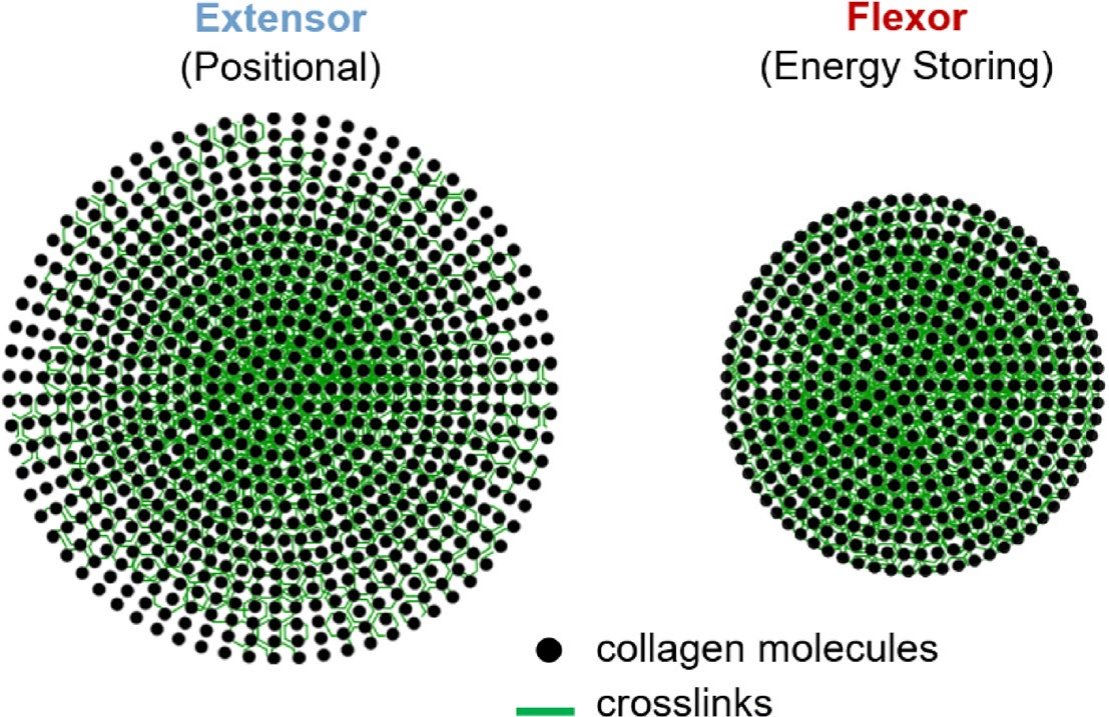
Simplified depictions of the cross-section of a collagen fibril from a bovine digital extensor tendon and from a superficial digital flexor tendon. Collagen fibrils from high-stress, energy-storing superficial digital flexor tendons are known to be smaller in diameter and more heavily crosslinked than those from low-stress, positional extensor tendons. We hypothesize that: (i) fibrils from both tendons have a more densely crosslinked core with a less crosslinked shell that decreases in crosslink density moving towards the fibril surface, (ii) that both fibril types contain similarly sized cores, (iii) that flexor fibrils have a thinner, but more crosslinked shell than extensor fibrils, and (iv) that enzymatically-controlled, intermolecular crosslinking is protective against fibril enzymolysis by MMP-1. Fibrils structured in this manner would explain why larger diameter fibrils showed greater susceptibility to enzymolysis by MMP-1, why small diameter flexor fibrils were resistant to MMP-1 enzymolysis, and why fibril populations from both tendon types converged to similar fibril diameter populations on exposure to MMP-1 (Fig. 1E).

Flexor fibrils may also have a greater crosslinked core compared to molecules on the periphery, or the shell of the fibril. One of the most interesting observations of the current study is that diameter distributions of the flexor and extensor fibrils after incubation were identical. Perhaps degradation approached a limit as the shell of both fibril types was removed, taking much longer in the more crosslinked but thinner shell of the flexor fibrils, leaving behind the more heavily crosslinked core possibly present at birth. An illustration of this core–shell structure in both fibril types can be seen in Fig. 12. The idea of a core–shell structure of collagen fibrils has been suggested by other work in the literature^9,37–41^. Mechanically, there is evidence for a core–shell structure in the phenotype of plastic damage reported for the same bovine flexor and extensor fibrils^5,9,11^. First discovered in positional fibrils like those from the tail^37,42,43^ and digital extensor tendons^5,9^, fibrils develop a damage motif termed discrete plasticity, characterized by regular kink damage visible on the surface of fibrils. However, using techniques such as AFM and second harmonic generation (SHG) microscopy, these damaged fibrils are shown to have a loose shell of denatured collagen that lacks D-banding, able to be degraded with the gelatinase MMP-9, while a more dense core that retains the D-band structure is found within^9,37,38^, consistent with earlier work^42,44^. While flexor fibrils show increased resistance to this plastic damage, this same damage motif was able to be created by decreasing strain rate^11^. This would support a core–shell even within the flexor fibrils, one that is more resistant to damage than the extensor fibrils due to the increased crosslinking. Other studies have also theorized a core–shell structure, though not always due to crosslinking^39–41^.

### *Relevance to *in vivo* tendon homeostasis*

Studies looking at collagen turnover in tendon show conflicting results. Some studies claim that collagen is not replaced after skeletal maturity^45,46^. Others have reported half-lives of 30–250 years^24^, 1.5–3 years^47^, and 2–5 weeks^48,49^, while a recent study provided compelling evidence for collagen synthesis and degradation following the 24 h circadian cycle^50^. These discrepancies are partially due to differing methodologies (e.g. technique and species), but may also be explained by the presence of two distinct pools of collagen in a tendon: a large ‘persistent’ pool making up the bulk of the tendon, and a smaller ‘sacrificial’ pool of newly synthesized collagen that can aid with remodelling and repair^50–52^.

The idea of a rapidly synthesized and degraded pool of collagen aligns well with current ideas regarding the mechanochemical sensitivity of collagen fibrils^17^. The structure of a collagen fibril enables it to influence its own assembly, growth, and degradation; and this structure is dynamically-responsive to applied strain^17^. Cells also respond to mechanical cues by altering their synthesis, modification, and secretion of collagen molecules, enzymes, and inhibitors. Because tendons have a low cell density, especially within the fascicular matrix, these mechanical cues may be more helpful and detectable than merely chemical ones.

The experimental conditions in the present study have some limitations when compared to in vivo conditions. The concentration of MMP-1 relative to collagen is far in excess (potentially two to three fold higher) of what has been reported in tissue^53^. The choice of MMP-1 concentration was largely practical. Based on the slower activity observed in our previous study^21^ we wanted to maximize the chances of detecting a difference if there was one. A lower concentration of MMP-1 may have prevented us from detecting any differences, particularly for the more degradation resistant flexor tendon. Similarly, the choice of using MMP-1 in isolation—without other enzymes or cofactors—simplifies the in vivo condition, but was necessary for this initial examination.

Addition of gelatinases, stromelysins, or even cathepsins may have facilitated degradation through removal of proteoglycans (PGs), other fibril associated proteins, or even collagen’s C-telopeptide^33^. However, the extent to which the addition of enzymes to remove these structures would have resulted in differences between the two tendon types is unclear. Proteoglycans may inhibit MMP-1 degradation^54^, but the bulk tendon and potential fascicular level differences in PG content between these tendons^25,30^ have not been resolved at the fibril level. Additionally, the extensive rinsing in our protocol prior to enzyme incubation likely removed some extrafibrillar proteins^55^. Cathepsin K may be a viable modulator of MMP-1 activity in vivo as it can cleave intact fibrils, including their telopeptides, and it is elevated in damaged tendon^56^. Removal of the C-telopeptide was thought to be necessary for MMP-1 access and subsequent cleavage^33^, but this was not required in our study. Additionally, Cathepsin K requires an acidic pH and GAGs to complex with in order to be active^56,57^. This is thought to occur within or in close proximity to fibroblast cells^56^. Our protocol lacked viable cells (due to rapid freezing) and occurred at a neutral pH, so Cathepsin K’s activity in our experimental conditions and subsequent contribution to changes in degradation would have been limited.

Perhaps the largest limitation of the current study was the absence of applied strain. This may have artificially enhanced the activity of MMP-1, with nominal to moderate applied strain being known to inhibit enzymatic degradation by either altering the MMP-1 cleavage site (refolding the partially unfolded α2-chain) or inhibiting access of the enzyme^13–16^. Lack of tension could also have decreased fibril density, and to some extent molecular packing in conjunction with swelling that can occur in PBS buffers^58^. Interestingly, the adherence of collagen fibrils to the glass substrate in our previous study^21^ may have maintained some level of pre-strain on the fibrils during incubation, potentially contributing to the slower degradation rate observed compared to that seen in the present work.

Whether the structural differences between the energy-storing and positional tendons result in altered degradation patterns with the addition of strain, or the removal of PGs or C-telopeptides, are areas for future research that will be aided by this investigation. The current results contribute to what is presently a small body of literature examining enzymatic degradation of collagen fibrils. Better understanding of factors that influence collagen fibril remodeling in tendon are needed to inform new studies and the design of new therapies and injury prevention strategies.

## Conclusion

This work offers a direct validation of previous work performed on isolated collagen fibrils by expanding our measurement of direct structural changes caused by degradation to the full fibril population within tendons. We have confirmed that the ability for MMP-1 to degrade collagen fibrils from functionally distinct tendons is not the same, likely owing to their differing structures, particularly molecular crosslinking. Low-stress, positional extensor fibrils are able to be degraded to a much larger extent than the more crosslinked fibrils from high-stress, energy-storing flexor tendons. Additionally, the relationship between fibril size and degradation was confirmed as larger fibrils were degraded more than smaller fibrils at a rate unexplained by differences in surface area alone. We postulate that this is due to the existence of a common more-crosslinked core. This hypothesis needs to be tested further, however if validated it would have a major impact on our understanding of tendon development and turnover. A first step could be to conduct crosslink density^5,43,59^ and/or chemical species analysis^36^ on degraded fibrils to compare the remaining core to native fibrils of the same type. There are currently no tools able to give information on the presence of individual crosslinks in a fibril, while simultaneously measuring other features/responses. Until methods are developed to do so, careful studies controlling for tissue sources and fibril diameter need to be implemented.

## Methods

### Theoretical modeling of degradation in a tendon section

#### Degradation models

**Fig. 13:**
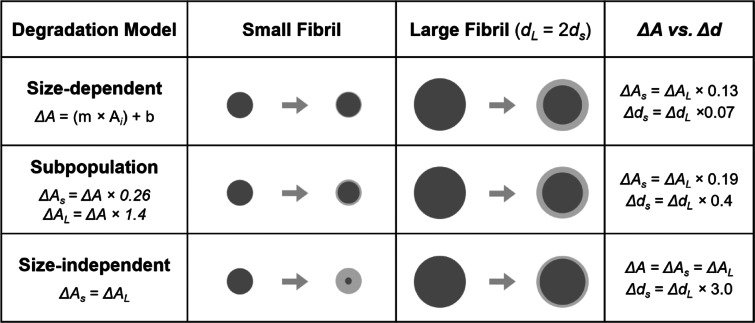
Example of how two fibrils with different diameters (small diameter, *d*_*s*_, and large diameter, *d*_*L*_) change dimension with the three different degradation models that vary the influence of fibril size (cross-sectional area, *A*) on degradation. Lighter grey indicates fibril area that is removed.

Enzyme degradation of collagen fibrils at the population level was modeled to inform experimental incubation length and explore the relationship of fibril size with degradation reported in our previous study^21^. Using the change in CSA (*∆CSA)* measured for individual collagen fibrils following 5 h of incubation^21^, three predictive models of degradation were created, depicted in Fig. 13. The first model**,** referred to as the size-dependent model, was a cross-sectional area dependent degradation model, and the model that best represented the degradation observed in Gsell et al.^21^. The linear equation for *∆CSA* was dependent on initial CSA (*CSA*_*i*_) for the bovine extensor fibrils, and was applied to *CSA*_*i*_ values calculated from a reference dataset of 416 fibril diameters from four bovine extensor tendons^5^. This yielded a unique *∆CSA* for each fibril:

$$\Delta CSA = - 65.94 + \left( {0.0721*CSA_{i} } \right)$$

The second model, referred to as the subpopulation model, was based on cross-sectional degradation with two different subpopulation dependent rates. Fibrils from the reference distribution were split into small (S1) and large (S2) diameter subpopulations via k-means clustering. Some researchers have suggested there are multiple populations of fibrils within a tendon, distinguishing these based on size as well as potential functional differences^22,50,60,61^, possibly resulting in the size effect observed in our previous study^21^. Using the *∆CSA* from the size-dependent model, an average *∆CSA* was calculated for each subpopulation and applied to the fibrils in that subpopulation only. The third model, referred to as the size-independent model, was based on cross-sectional degradation at a constant rate. This model used the *∆CSA* from the size-dependent model to calculate one average *∆CSA* to be applied to the entire population of fibrils.

For each model, incubation was modeled in 5 h increments up to 25 h of incubation. For the size-dependent model, the individual *∆CSA* for each fibril was recalculated for every 5 h increment, while for the subpopulation and size-independent models, the same average *∆CSAs* from the initial calculations were used for each 5 h extrapolation. The population CSAs at each time point were reported in addition to the absolute and cumulative change in diameter for each incubation interval. Distributions for 24 h of enzyme incubation were also calculated to match the incubation time conducted in the subsequent degradation experiment. This was done by multiplying the *∆CSA* between 20 and 25 h by 4/5^th^ and subtracting from the 20 h CSA.

#### Minimum incubation time calculation

A priori power analyses of the full reference dataset and its two subpopulations (S1 and S2) were calculated using the mean, standard deviation (SD), and number of samples (N) for each. This was based on one-sided, 2-sample t-tests to determine the minimum effect sizes needed to detect a difference between two distributions of fibril diameter. The average cumulative decrease in fibril diameter for each population corresponding to the calculated minimum effect sizes were then interpolated to determine the number of hours (rounded up to a whole number) of incubation needed to achieve this.

Analysis of the full distribution produced an effect size of 8.6 nm and 11.4 nm for 80% and 95% power, respectively. For the more conservative 95% power, the number of hours of incubation required to achieve this was between 8 and 13 h depending on the degradation model. The size-independent degradation model required the least amount of time, while the size-dependent model required the most. In order to detect changes in diameter at the subpopulation level, the longest incubation time required was 20 h for the small fibril diameter subpopulation with the size-dependent model (95% power). Complete results are shown in Table 1.

**Table 1:** Descriptive statistics of the collagen fibril diameter distribution and subpopulation (S1 and S2) distributions from reference dataset^5^ and minimum effect size calculated via a power analysis.

Data	Mean (nm)	SD (nm)	N	80% Power				95% Power			
				Min Effect Size (nm)	Size-dependent	Sub-population	Size-independent	Min Effect Size (nm)	Size-dependent	Sub-population	Size-independent
					Incubation Required (hrs)				Incubation Required (hrs)		
Full	133.9	49.7	416	8.6	10	9	7	11.4	13	11	8
S1	75.9	21.1	148	6.1	15	12	4	8.1	20	15	8
S2	165.9	26.7	268	5.7	5	5	7	7.6	7	7	9

Hours of incubation are based on the cumulative decrease in fibril diameter modeled with size-dependent, subpopulation, and size-independent models of degradation.

### Experimental degradation of tendon sections

#### Sample preparation and storage

Tissue harvest was approved by the University Committee on Laboratory Animals at Dalhousie University. A bovine forelimb from an adult steer (2–3 years of age) was obtained from a local abattoir and dissected to isolate the paired lateral digital extensor (LDE) and superficial digital flexor (SDF) tendons. Tendons were immediately prepared for storage by removing the epitenon and blunt cut ends, dividing each tendon cross-sectionally then longitudinally to produce ~ 9 × 3 mm strips, placing each strip in 1 × 1 cm silicone wells containing optimal cutting temperature (OCT) compound, and then freezing via submersion in liquid nitrogen. This produced frozen tissue cubes (Figure S1) that were stored at − 80 °C until use to limit any fibril damage that may occur with multiple freeze–thaw cycles^62^.

#### Tissue sectioning and adherence

A single frozen tissue cube was cryosectioned into 100-μm-thick sections using a sliding microtome (Leica SM2000 R). Sections were immediately transferred into wells containing double distilled water (ddH_2_O) to dissolve the OCT surrounding the tendon sample. This was done using tweezers with care taken not to grip the tendon portion. The sections were then transferred into fresh wells containing phosphate-buffered saline (PBS) for each of three 20 min rinsing steps under agitation to remove any remaining OCT (critical for subsequent adhesion and degradation steps). Section transfers were done using a fresh round glass cover slide under the sample as a transfer surface to lift (with tweezers) then deposit the sample into a fresh well.

Once rinsed, sections were similarly transferred into wells of a pre-prepared adhesion plate (see Supplemental Material including Figure S2) filled with ddH_2_O. Briefly, this plate contained round glass cover slides coated with Cell-Tak (Corning® Cell-Tak CLS354240, 1.26 mg/mL in acetic acid), a cell and tissue adhesive, and loosely adhered to the bottom of each well using polydimethylsiloxane (PDMS). The water was removed using a transfer pipette, ensuring full contact of the section with the adhesive target, then a drop was pipetted back onto the surface of the section to maintain hydration. After a minimum of 30 min the sections were considered adhered to the glass slide, which could be removed from the well using shear force applied with tweezers. This allowed for the slide and adhered sample to be transferred into fresh wells for further experimentation.

#### Enzyme incubation

Test sections were incubated in 2 mL of MMP buffer (100 mM Tris–HCl, 10 mM CaCl2, 100 mM NaCl, pH 7.5) with enzyme conditions containing 10 µg, equating to ~ 117 nM of MMP-1 (SRP3117, Sigma Aldrich, USA), while control sections were incubated in buffer alone. Unlike previous studies^21,63,64^, Zn acetate was not added to the buffer due to excessive precipitation of ZnO crystals out of solution, impeding visualization of fibrils (see Supplemental Material including Figure S3). Incubation was performed at 37 °C for 24 h under constant agitation at 2 Hz^21,38^. MMP-1 concentration and incubation time were chosen for maximal degradation potential within experimental limitations. The conservative 20 h incubation predicted from degradation modeling using data from our previous study was based only on extensor fibrils^21^. Because flexor fibrils showed no measurable degradation after 5 h in that study^21^, the MMP-1 concentration was increased in the present study and the incubation time was extended to 24 h to increase the likelihood of detecting degradation in the flexor fibrils, and to account for the higher collagen content in tendon sections. The experiment was not performed in sterile conditions which limited further extending the duration of incubation.

#### Sample fixation and preparation for SEM imaging

Following incubation, sections were fixed and dehydrated prior to imaging. Sections were transferred into wells containing 2.5% glutaraldehyde for 1 hr^5,42^, followed by two 15 min rinses in ddH_2_O, and 15 min exchanges in a graded ethanol series: 30, 70, 90, 95 and 100% EtOH, each under agitation on a shaker table. Sections were then transferred into histology tissue cassettes for critical point drying (Leica EM CPD 300).

Dried sections were carefully removed from their glass slides if not already detached during the dehydration procedure and mounted on SEM stubs with maintenance of the sample surface orientation. Samples were sputter coated with gold palladium (80%/20% by weight) (Leica EM ACE200, 70 s at 30 mA) and stored under vacuum desiccation until imaging.

#### SEM imaging and analysis

Scanning electron microscopy (SEM) (Zeiss Sigma 300 FE-SEM at 5 kV of accelerating voltage) was performed to obtain both high (~ 15kX) and low magnification images of the samples for quantitative and qualitative analyses, respectively. Quantitative image analysis was performed with a custom semi-automated pipeline in MATLAB (vR2022b, Mathworks, USA). Briefly, fibrils with both edges visible for a minimum of 2 μm were semi-manually segmented from SEM images, visible in Figs. 8 E, F and 9E, F, followed by automated piecewise edge detection along each fibril to account for segmentation inaccuracies (example shown in Figure S4).

Pieces 250 nm in length were chosen to best handle fibril curvature, while still remaining in the 200–1000 nm span of long-range structural variation inherent to collagen fibrils^65^. Piecewise analysis yielded the average and standard deviation of each fibril’s diameter and orientation. Fibril diameters from the control samples were converted to CSA and input into the 3 models of degradation described previously. This was done to compare the experimentally observed enzyme data with the modeled enzyme data to identify influence of fibril size on degradation that was previously reported^21^. Fibril alignment was calculated by normalizing the average piecewise fibril orientation angle to the average of each image. Fibril curvature was calculated as the cumulative absolute change in piecewise orientation angle normalized to distance in micrometers.

A separate analysis module was created to calculate the D-band length for each fibril using outputs from the piecewise analysis. Similar to D-Band analysis using height data from AFM images^21,37,66^, the fibril centerline calculated from the piecewise analysis was expanded by 2 pixels on either side to give an average longitudinal trace of the fibril. This 5-pixel-wide profile was smoothed using a moving average filter, zeroed by subtraction of the filtered trace, then interpolated to one greater than the nearest power of 2 datapoints. A fast Fourier transform (FFT) was used to analyze the power density spectrum to determine the spatial frequency with maximum power corresponding to the D-band. The region within which to locate the D-band peak was constrained to between 36 and 70 nm based on iterative optimization. The full fibril length was used for D-band analysis rather than a piecewise analysis to increase the signal-to-noise ratio (SNR) as differences in pixel intensity were small, particularly in the enzyme treated samples. Due to large variations in D-band lengths calculated in this study and previous pilot data, partially due to automated maximum peak selection, the power spectra for all the fibrils in each sample were averaged to enhance SNR further. Finding the maximum peak within the D-band range from the averaged power spectra was much more precise than peak detection in individual fibril power spectra.

#### Statistical analysis

JMP® statistical software (v18.2.1 (Student Ed.), SAS Institute, USA) with an alpha level of 0.05 was used for all tests. Normality of data was assessed using Shapiro–Wilk tests. Nonparametric tests were used for all comparisons with the exception of fibril alignment due to the presence of non-normal distributions.

Mean fibril diameter comparisons between the full distributions of fibril diameters were done using Wilcoxon tests (one per tendon) and the subpopulation distributions were assessed using a Kruskal–Wallis test with post hoc comparisons made using the Dunn (all pairs) method for joint ranking. Additionally, subpopulation diameters from the flexor and extensor tendon reference dataset from Herod et al.^5^ were compared with the control subpopulations from the current study using the same methods.

Variation in fibril diameter and fibril D-band length were compared with tendon type and incubation condition as factors using a 2-way ANOVA with rank-transformed data to handle non-normality. Post hoc comparisons were made Tukey’s adjustment. To assess the impact of fibril diameter as a covariate, ANCOVAs were conducted on raw enzyme data for both diameter variation and D-band length, with the effect of tendon, fibril diameter, and their interaction examined. Control data was not assessed this way due to non-linearity of the outcome measure with fibril diameter, as all 4 samples were evaluated for linearity using simple linear regressions.

The variance in fibril alignment was compared between all 4 samples using O’Brien’s F-test with individual comparisons made using two-sample F-tests with Bonferroni correction. Fibril curvature was compared for all 4 samples using a Kruskal–Wallis test with post hoc comparisons made using the Dunn (all pairs) method for joint ranking.

## Supplementary Information

Below is the link to the electronic supplementary material.

Supplementary Material 1

## Data Availability

Data sets generated during the current study are available from the corresponding author on request. MATLAB code for the custom image analysis pipeline constructed for this study is available at: https://github.com/kreplak-research-group/.github
