# Supplementary material for: In tendons, differing physiological requirements lead to distinct patterns of MMP-1 degradation
Source: Sci Rep. 2025 Dec 23;16:2420. doi: 10.1038/s41598-025-32374-3 (PMC12820382; doi:10.1038/s41598-025-32374-3)
Supplement: Supplementary file 1 — Supplementary Material 1 [file 41598_2025_32374_MOESM1_ESM.pdf]

SUPPLEMENTARY MATERIAL

In tendons, differing physiological requirements lead to distinct patterns of MMP-1 degradation

Kelsey Y. Gsell[a], Laurent Kreplak[a,b], and Samuel P. Veres[a,c,*]

[a] School of Biomedical Engineering, Dalhousie University, Halifax, Nova Scotia, Canada

[b] Physics and Atmospheric Science, Dalhousie University, Halifax, Nova Scotia, Canada

[c] Division of Engineering, Saint Mary's University, Halifax, Nova Scotia, Canada

*Corresponding author.

Email addresses: sam.veres@smu.ca (SP Veres)

1. Tissue dissection and storage

Figure S1 provides a visual representation of the steps involved in the preparation of tendon strips for cryosectioning, including dissection of the tendon and method of freezing.

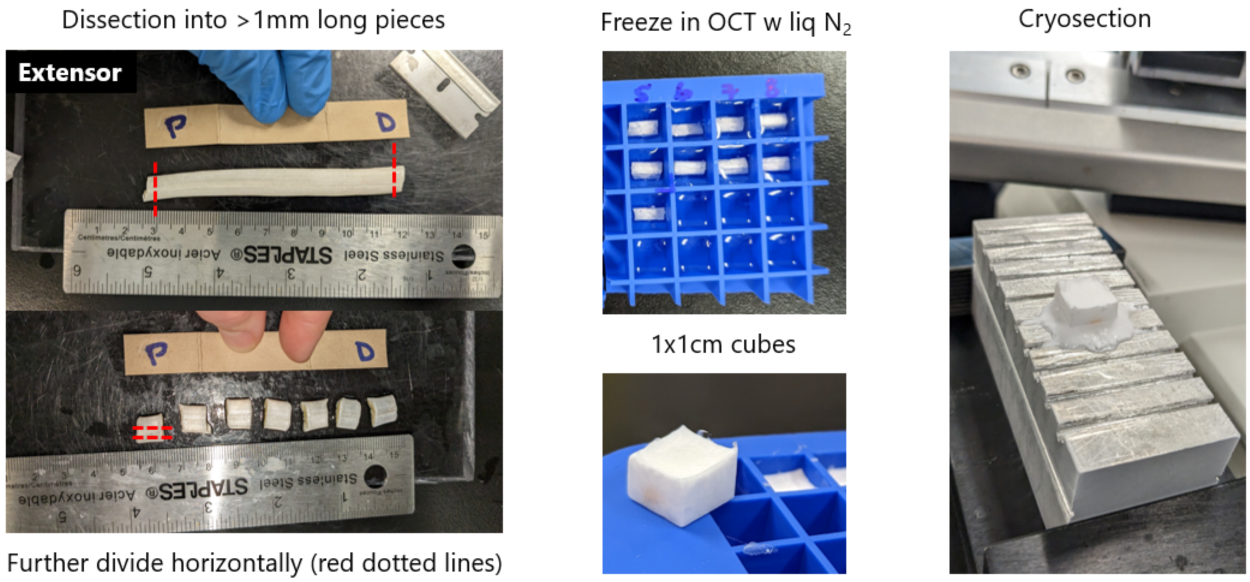

Figure S1 - Tendon preparation for storage and subsequent cryosectioning.

2. Adhesion Plate Preparation

A 24-well plate was prepared to enable sample adhesion and transfer required for the final experimental protocol (Figure S2). A pin-prick amount of liquid (uncured) polydimethylsiloxane (PDMS) was lightly touched to the center of a well with a 10 µL pipette tip, and a round glass cover slide (thickness #2 (0.17-0.25 mm), 12 mm diameter) was placed on top. Once cured, this secured the glass slide to the bottom of the well. The PDMS was mixed in an 11:1 ratio of base to curing agent (Sylgard® 184 Silicone Elastomer Kit, DOW 19J3507) to produce a softer PDMS allowing for subsequent detachment and transfer later in the protocol. A cell and tissue adhesive, Corning® Cell-Tak (CLS354240, 1.26 mg/mL in acetic acid), was coated onto the secured glass slide using the hand-spreading and adsorption method provided by

the manufacturer. A 10 μL drop of Cell-Tak was pipetted into the center of the glass slide and spread into a rectangular shape using the pipette tip. The plate was then put into a biological safety cabinet (BSC) to allow for evaporation of the solution resulting in an adhesive layer left on the glass. Once fully dry, the slides were rinsed with 70% ethanol (EtOH) then water as per the manufacturer's instructions. Once dry, the plates were stored until ready for use.

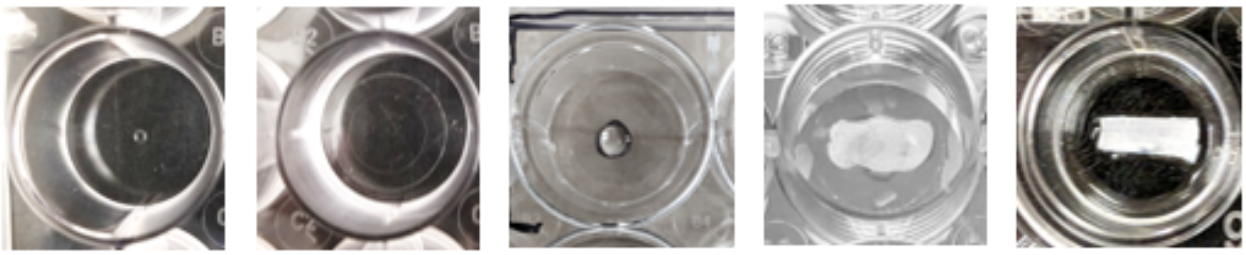

Figure S2 - Preparation of well-plates for sample adhesion and transfer. From left to right: pin sized amount of uncured PDMS, round glass cover slide adhered to well, drop of Cell-Tak tissue adhesive, Cell-Talk after being spread and evaporated, tendon section adhered to glass slide via Cell-Tak.

Adhesion of the sample to a glass slide was necessary for solution changes during the experiment, both to make transfers easy and to ensure the orientation and full submersion of the section was maintained, and to move the sample without direct contact to avoid inflicting damage and having the section curl or fold.

3. Pilot MMP-1 degradation with Zn Acetate in Buffer

Bovine lateral digital extensor tendon sections were incubated in MMP buffer alone (control) or buffer containing 10 μg of MMP-1 (enzyme) (SRP3117, Sigma Aldrich, USA) for two consecutive 24 hr incubations, with fresh solutions used for each incubation period. Incubation was conducted at 37°C under constant agitation (2 Hz). MMP buffer contained 100 mM Tris-HCl, 10 mM CaCl2, 100 mM NaCl, and 2 μM Zn Acetate [$Zn(CH_3CO_2)_2$] at pH 7.5[1–3].

Following sample fixation and preparation for imaging, as described in Section 5.2.4, samples were imaged with scanning electron microscopy (SEM) as described in Section 5.2.5, in addition to energy dispersive x-ray spectroscopy (EDS) performed using a MIRA3 FE-SEM at 20 kV of accelerating voltage to determine the presence of atomic elements.

The presence of Zn acetate in the incubation buffer resulted in high contrast crystal-like deposits on the surface of the tendon section, only when incubated with MMP-1. Examples are shown in Figure S3A&B along with a comparable Zn structure reported in the literature: ZnO formed through the thermal decomposition of Zn carbonate (Figure S3D)[4]. EDS analysis was conducted for 3 regions within the crystal deposit (indicated on Figure S3C). The composition of the elements present in the sample reported as the mean and standard deviation of percent weight were as follows: 26.2 ± 1.0% of carbon, 2.6 ± 0.3% of nitrogen, 36.6 ± 0.8% of oxygen, 4.1 ± 0.2% of sodium, 7.1 ± 0.1% of phosphorus, 0.06 ± 0.5% of chlorine, 3.3 ± 0.1% of calcium, and 20.1 ± 0.2% of zinc. These results suggest the deposits observed in our experiment are ZnO nanoparticles.

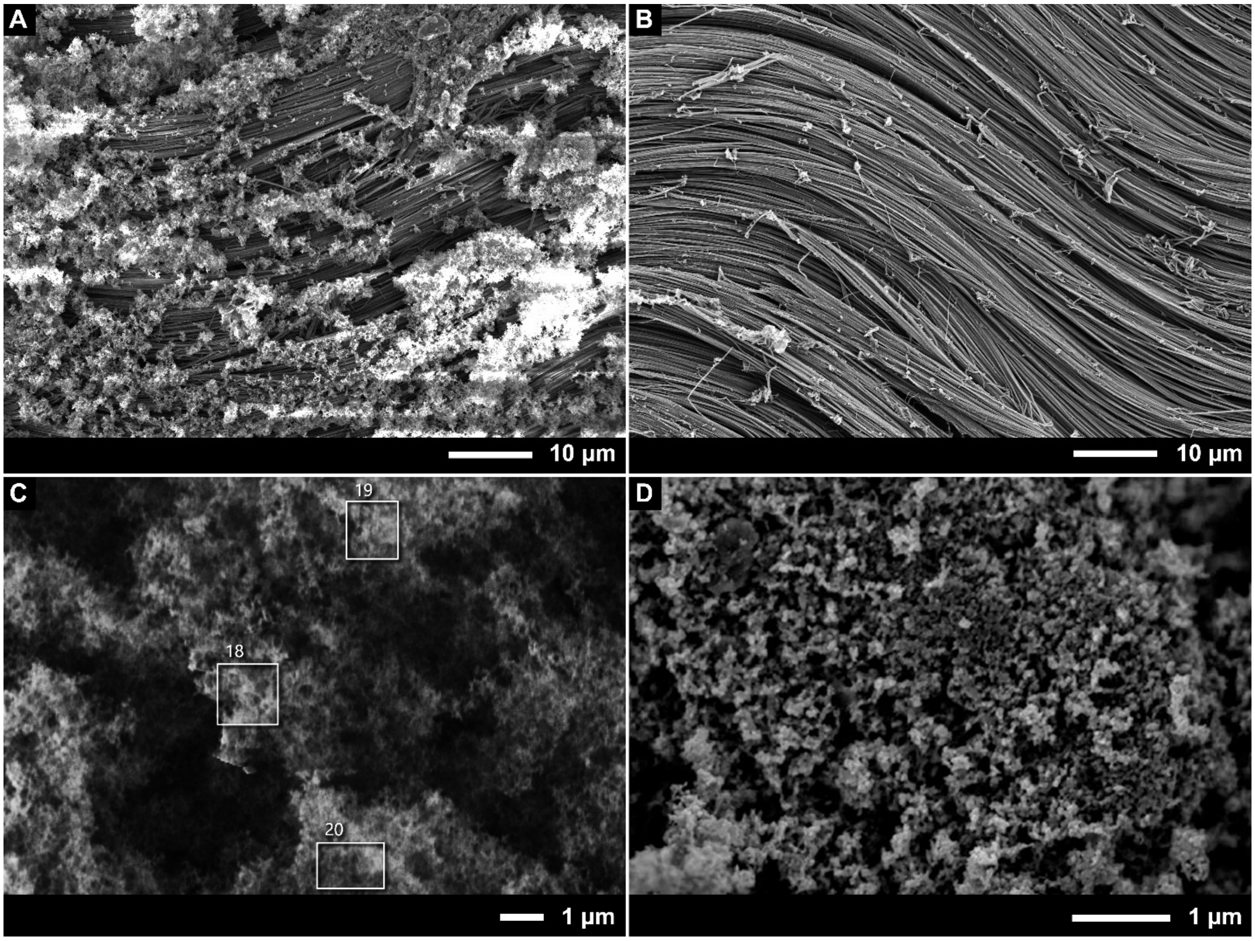

Figure S3 – **A)** Crystal deposits were seen in SEM images of extensor sections incubated in MMP buffer containing Zn acetate in the presence of MMP-1, but not when MMP-1 was absent **(B)**. **C)** Three crystallized regions in the enzyme treated sample from A were used for EDS analysis. **D)** ZnO nanoparticle structure reported in[4].

4. Piece-wise fibril diameter analysis

Figure S4 gives an example of a segmented fibril that has automated piece-wise edge detection performed on it. The fibril is split into 250nm long pieces (Figure S4A), each of which is rotated with the fibril oriented vertically to enable edge detection (shown with dashed green lines) (Figure S4B). Detailed explanation of the pipeline can be found in[5], with MATLAB code available at: https://github.com/kreplak-research-group/SEM-Image-Analysis-of-Collagen-Fibrils.

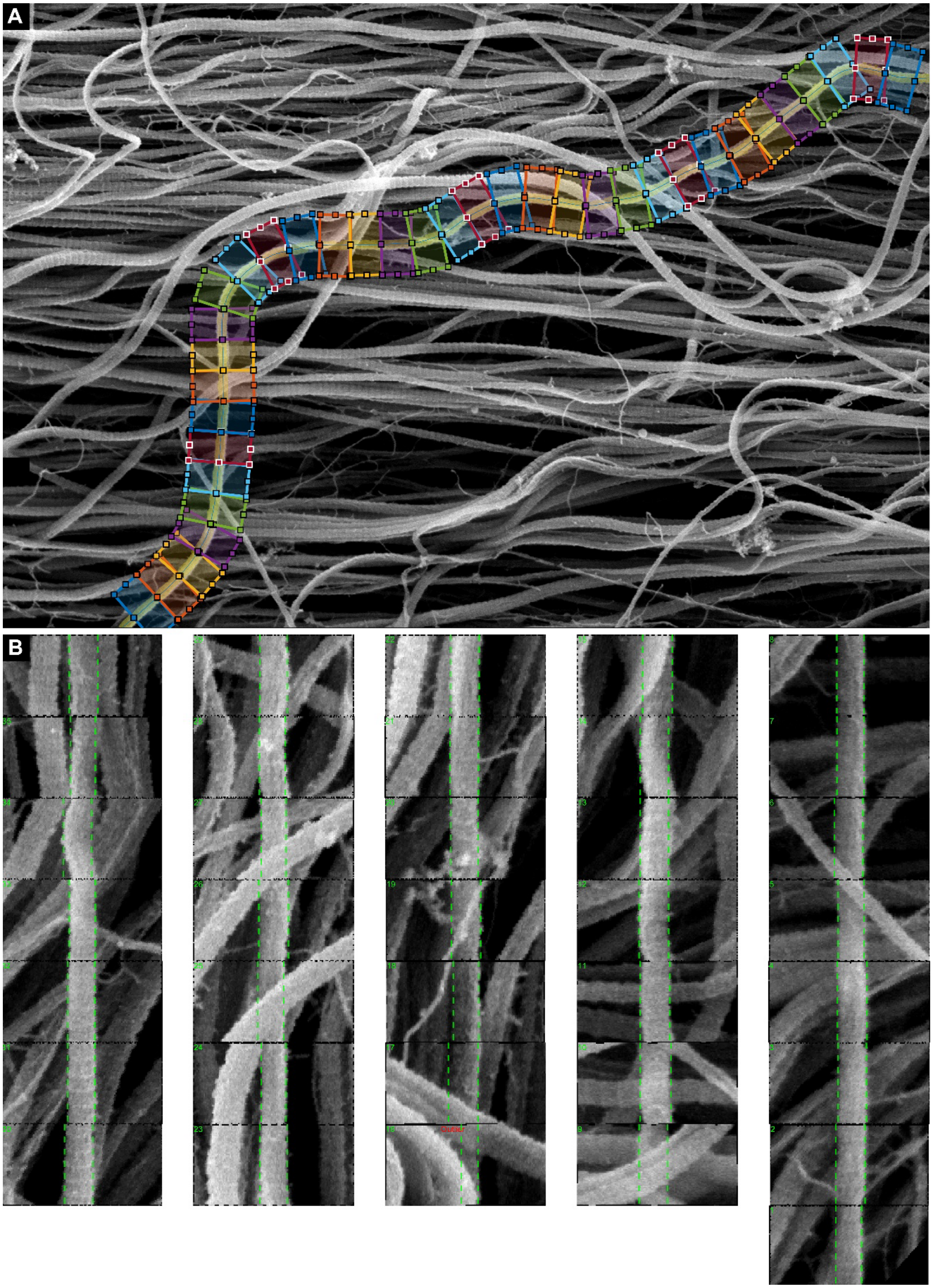

Figure S4 - Example of a fibril imaged from a tendon section (flexor incubated with MMP-1) that had automated piecewise analysis performed to calculate average fibril diameter. **A)** Fibril was split into 250 nm long pieces (500 nm width) with **B)** subsequent piece-wise fibril edge detection. There are 36 pieces, with piece 1 starting in the bottom left corner in (A), but bottom right corner in (B).

References

1. K.Y. Gsell, S.P. Veres, L. Kreplak, Single collagen fibrils isolated from high stress and low stress tendons show differing susceptibility to enzymatic degradation by the interstitial collagenase matrix metalloproteinase-1 (MMP-1), Matrix Biol Plus 18 (2023) 100129. https://doi.org/10.1016/j.mbplus.2023.100129.
2. S.S. Veidal, M.A. Karsdal, E. Vassiliadis, A. Nawrocki, M.R. Larsen, Q.H.T. Nguyen, P. Hägglund, Y. Luo, Q. Zheng, B. Vainer, D.J. Leeming, MMP Mediated Degradation of Type VI Collagen Is Highly Associated with Liver Fibrosis – Identification and Validation of a Novel Biochemical Marker Assay, PLOS ONE 6 (2011) e24753. https://doi.org/10.1371/journal.pone.0024753.
3. J. Sikora, M. Cyrankiewicz, T. Wybranowski, B. Ziomkowska, B. Ośmiałowski, E. Obońska, B. Augustyńska, S. Kruszewski, J. Kubica, Use of time-resolved fluorescence spectroscopy to evaluate diagnostic value of collagen degradation products, J Biomed Opt 20 (2015) 051039. https://doi.org/10.1117/1.JBO.20.5.051039.
4. T. Tokarski, E. Olejnik, B. Hutera, A. Kmita, Synthesis of Zno Nanoparticles by Thermal Decomposition of Basic Zinc Carbonate, Archives of Metallurgy and Materials; 2013; No 2 June (2013). https://journals.pan.pl/dlibra/publication/101428/edition/87445 (accessed September 13, 2024).
5. K. Gsell, Collagen fibrils from functionally distinct tendons have differing susceptibility to degradation by MMP-1, (2024). https://hdl.handle.net/10222/84771 (accessed June 5, 2025).
